# Vikrahraun—the 1961 basaltic lava flow eruption at Askja, Iceland: morphology, geochemistry, and planetary analogs

**DOI:** 10.1186/s40623-022-01711-5

**Published:** 2022-11-12

**Authors:** Aline Y. Blasizzo, Ingrid A. Ukstins, Stephen P. Scheidt, Alison H. Graettinger, David W. Peate, Tamara L. Carley, Adam J. Moritz, Jennifer E. Thines

**Affiliations:** 1 The University of Iowa, Iowa City, IA 52242 USA; 2 The University of Auckland, Private Bag 92019, Auckland, 1142 New Zealand; 3 Howard University, 2400 6th St NW, Washington, DC 20059 USA; 4 University of Missouri-Kansas City, 5000 Holmes St, Kansas City, MO 64110 USA; 5 Lafayette College, 730 High St, Easton, PA 18042 USA; 6 New Mexico State University, 1780 E University Ave, Las Cruces, NM 88003 USA

**Keywords:** Askja, Iceland, Morphology, Basalt textures, Planetary analogs, Geochemistry, Remote sensing

## Abstract

We examine the morphology and chemistry of the Vikrahraun basaltic eruption emplaced at Askja Volcano, Iceland, from Oct. 26–Dec. 17, 1961. The eruption had three eruptive events, initiating with aʻa and followed by alternating aʻa and pahoehoe lava flow emplacement. We determine that while the eruption is chemically homogenous (Fe/Mg = 1.9–2.2, 47–52 wt.% SiO2), it demonstrates transitions from high to low viscosity lava flow morphologies. Aʻa flows have a total crystallinity (phenocryst and microlite abundance by area) ranging from 85–100%, which increases by 1% per km from the vents, while pahoehoe flows range from 55–86% and increase at a higher rate of 5% per km. Vesicularity systematically decreases with distance from the vent by 3% per km. Pahoehoe and vent samples have calculated temperatures 50 °C higher than aʻa samples, which we interpret to be due to the difference between tube fed pahoehoe and open channel aʻa lavas. The homogenous nature of the Vikrahraun lava makes it an excellent testbed to study the effects of observational scale and satellite resolution on the interpretation of surficial textures. Festoons, which are downslope pointed convex ridges from 1 to 5 m high and ~ 10 m long, are observed in event 2 aʻa lavas in satellite imagery and topographic profiles. Features of this scale have previously only been documented in terrestrial rhyolitic lavas, leading planetary researchers to infer that festooned lava flows on Venus and Mars may be silicic. The diverse morphologies and homogenous composition make Vikrahraun an important planetary analog, where morphological complexity is over-attributed to chemical variation and suggests the need to re-evaluation planetary lava flow interpretations.

**Graphical Abstract:**

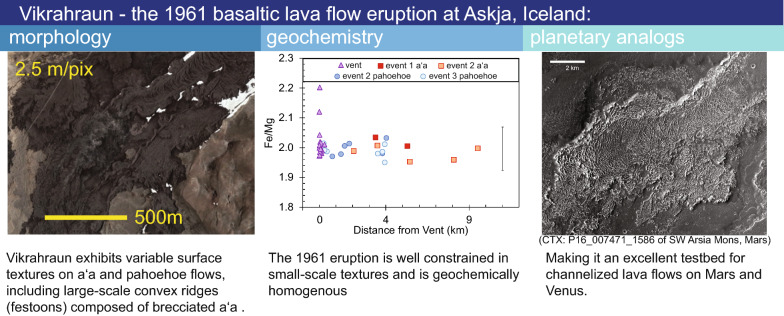

**Supplementary Information:**

The online version contains supplementary material available at 10.1186/s40623-022-01711-5.

## Main text

## Introduction

Studies of terrestrial lava flows as analogs to extra-terrestrial volcanic features are an essential component of planetary geology (Evans 1978; Fink 1980; Theilig and Greeley 1986, 1987; Garry et al. 2007; Wroblewski et al. 2019). A traditional method of estimating the viscosity and emplacement dynamics of planetary lava flows uses dimensional analyses of flow lobes and surface features (Fink 1980; Theilig and Greeley 1986, 1987; Garry et al. 2007). However, due to the nature of satellite data, these studies provide little insight into the internal structure or compositions (i.e., vesicularity, crystallinity, chemistry), which are known to affect the rheological properties of a flow system along with external factors such as effusion rates, temperature, and underlying topography (Peterson and Tilling 1980; Rowland and Walker 1990; Harris et al. 2007; Sehlke et al. 2014; Robert et al. 2014; Soldati et al. 2018). By calibrating these attributes using in situ measurements on a terrestrial lava testbed, more robust interpretations of emplacement dynamics of planetary lava flows are possible.

The 1961 basaltic lava flow of Askja, Iceland, named Vikrahraun, is an excellent candidate for a testbed study quantifying conditions impacting small and large-scale textures. Vikrahraun had near-continuous observation and documentation of the eruption effusion rates, advancement speeds, and flow lobe dimensions (Thorarinsson and Sigvaldason 1962; Thorarinsson 1963). The Vikrahraun lava flow also has one of the closest compositions to Martian basalts found on Earth, with Fe/Mg of about 1.9–2.2, which is comparable to values determined by gamma ray spectroscopy for Mars lavas (Fe/Mg ratios of 1.5–4) (McSween et al. 2009). In comparison, other flows from the Askja Volcanic system from the twentieth century and the 2015 Holuhraun eruption have lower Fe/Mg ratios of 1.0—1.3 (Hartley and Thordarson 2013; Geiger et al. 2016). Nýjahraun, in the North Volcanic Zone, has a recorded maximum Fe/Mg of 2.0 (Hartley and Thordarson 2013) (Additional file 5: Fig. S1). The 1961 lava flow is also distinguishable from the underlying topography in satellite imagery with minimal vegetation, making its areal extent well defined for satellite-based research. Additionally, the high latitude position of the Icelandic Highlands creates an analogous setting to the glaciovolcanic environment of Mars (Cousins et al. 2013).

This study presents the most extensive analysis of the 1961 Vikrahraun eruption to date. We link mineral chemistry, whole-rock chemistry, crystallinity, vesicularity, and density to observed surface pahoehoe and aʻa textures and larger scale features captured by topographic profiles, aerial photos, drone, and satellite imagery. These datasets are combined with an observational record of the eruption (Thorarinsson and Sigvaldason 1962; Thorarinsson 1963) in order to reinterpret the progression of flow emplacement. We dissect the flow system into its architectural components to explore the relationship between satellite interpretation and ground observations. We discuss the implications satellite-only interpretation has towards planetary lava composition in order to promote better understanding of terrestrial analogs for and planetary sciences.

### Geologic background

**Fig. 1:**
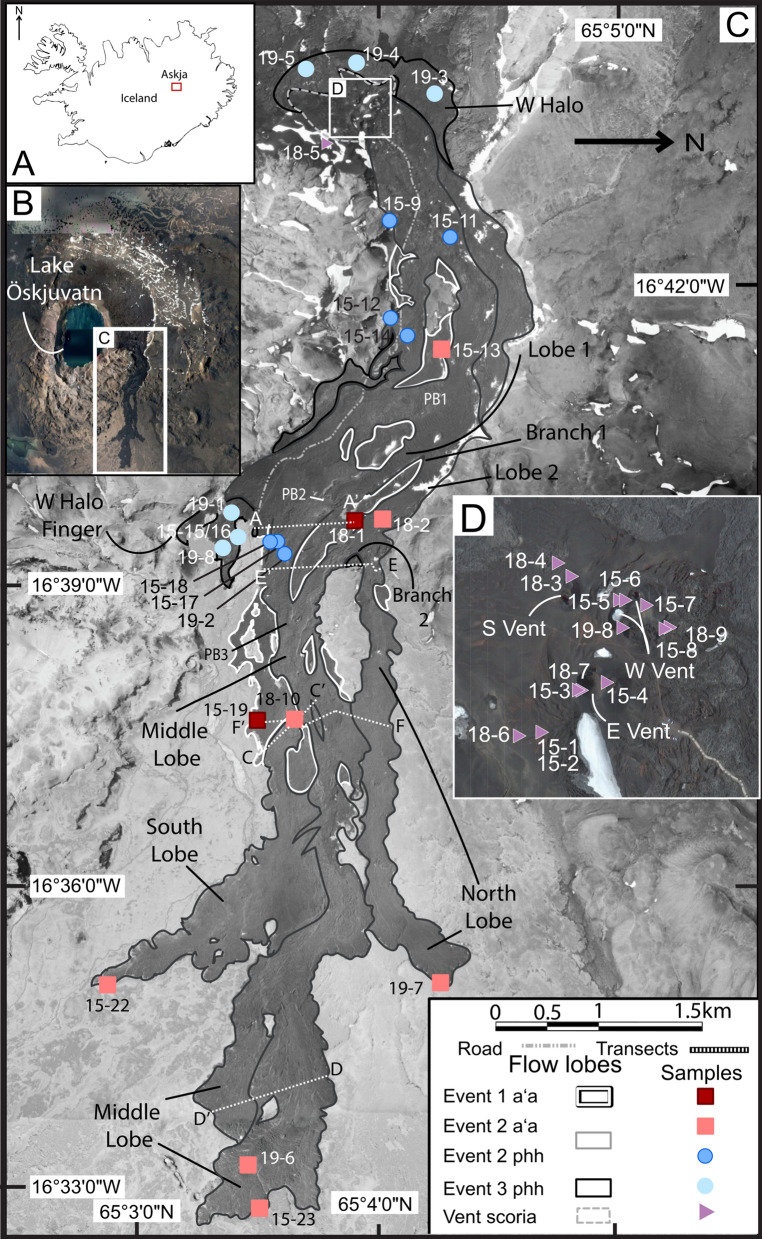
**A** Map of Iceland showing the location of Askja (red box). **B** CNES Airbus satellite image from Google Earth of Askja volcano, with Lake Öskjuvatn and the 1961 lava flow within the white box. **C** Greyscale aerial photo (taken by Loftmyndir ehf) (1 m/pixel) of the 1961 lava flow showing outlines of flow lobes from each eruptive event, differentiating predominant lava texture type (aʻa or pahoehoe). ‘phh’ = pahoehoe; ‘PB’ = pahoehoe breakout; “Branch” = locations where flow bifurcates. Locations of topographic profiles and samples are shown, sample names are abbreviated to collection year and sample number. **D** SPOT satellite imagery (2.5 m/pixel) displaying sample locations at the vents

Askja is a central volcano located in the highlands of Iceland in the Northern Volcanic Zone (Fig. 1a). It is surrounded by palagonite tuffs, breccias and basaltic lavas of the Pleistocene Dyngjufjöll Massif (Fig. 1b) (Thorarinsson and Sigvaldason 1962; Evans 1978; Hartley and Thordarson 2012; Graettinger et al. 2013). Askja is volcanically active, with several eruptions occurring in the nineteenth—twentieth century (Sparks et al. 1977; Sigurdsson and Sparks 1981; Hartley and Thordarson 2013). The largest of these eruptions was a caldera collapse event in 1875, which created lake Öskjuvatn and was of a compositionally heterogeneous mixture of basaltic and rhyolitic magmas (SiO_2_ compositions of 51 wt.% and 74 wt.%: Sparks et al. 1977; Sigurdsson and Sparks 1981). The collapse event was followed in 1921 and 1961 by eruption of basaltic lavas (Thorarinsson and Sigvaldason 1962; Kuritani et al. 2011) and illustrates the compositional diversity of the Askja magmatic system (Sparks et al. 1977; Kuritani et al. 2011; Hartley and Thordarson 2013).

**Fig. 2:**
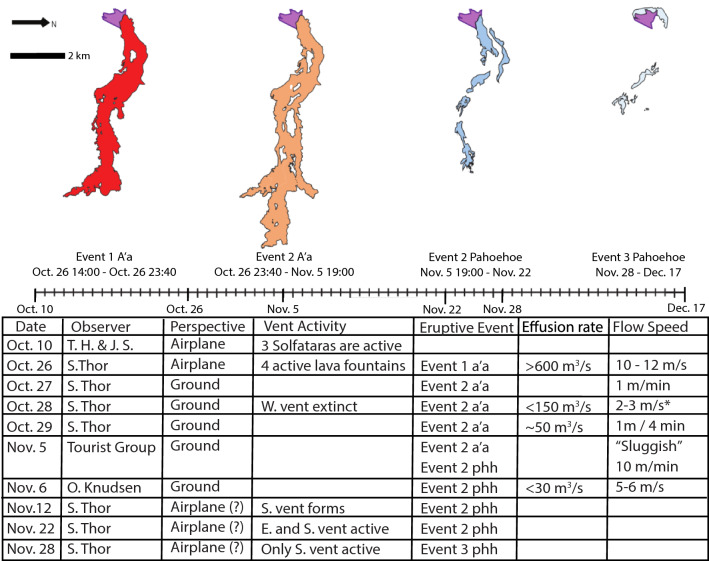
Eruption timeline, recording the date, the observer, the method of observation (airplane vs. ground), the emplacing event and texture, and when applicable, active vents (located in the purple polygon), effusion rates, and flow front speeds. The mapped areal emplacement and temporal stratigraphy interpretation is from this study. ‘S. Thor.’ = Sigurđur Thorarinsson, the primary investigator during the eruption (Thorarinsson, 1963). The asterisk indicates a localized observation of a lava river coming from the west vent

The 1961 eruption was originally documented by Thorarinsson and Sigvaldason (1962) who divided it into three eruptive sequences (summarized in Fig. 2), all of which initiated from lava fountains located on the northern margin of Lake Öskjuvatn (Fig. 1).

The first lava flow occurred on Oct. 26 with an aʻa that traveled 7.75 km east from the vents, which here is referred to as the “event 1 aʻa” (Fig. 1c and Additional file 5: Fig. S2). The second eruptive event initiated on Oct. 27 and was dominated by aʻa with volumetrically minor late-stage breakout pahoehoe channelized within the aʻa levees (Fig. 1c: “event 2 aʻa” and “event 2 pahoehoe”). The last phase was pahoehoe lava (“event 3 pahoehoe”), which flowed through tubes within the previously emplaced lava to the east and west of the vents on Nov. 28 (Fig. 1). There is uncertainty about when the eruption ended due to lack of direct observation, however it was officially declared ceased on Dec. 17, 1961.

From the vents to the flow front, the elevation of the pre-eruption surface decreases at an average of about 30 m/km (~ 1.7°) from west to east. However, this pre-eruption surface is not uniform and has localized hummocks of topographic highs as well as down-dropped ramps formed by underlying historic lava flow boundaries and NE–SW trending faults and lineaments (Hartley and Thordarson 2012). The influence of the underlying topography on the 1961 lava is best exemplified by the breakout lobes on the southern edge 5 km east of the vents, which can be seen flowing down and around topographically variable ramp slopes (Fig. 1c). Additionally, the presence of kipukas expose pre-eruptive topographic highs from the 1875 pumice (Hartley and Thordarson 2012). The largest of these kipukas is 4 km downflow (Fig. 1c), exhibiting stepped drops totaling 25 m across 1.1 km in length.

## Methods

Three field seasons were conducted in 2015, 2018, and 2019, where a total of 38 rock samples (15 vent scoria, 9 aʻa, 14 pahoehoe) (Table 1) and six topographic profiles (A-F) using Jacob’s staff were collected from the 1961 lava surface (Fig. 1c; Additional file 1: Sheets 1–2; Table 2; Additional file 4). In this study, we obtained thin section textures, bulk and matrix density, connected porosity, whole-rock chemistry, and mineral phase compositions of collected samples (Table 1; Additional file 1: Sheet 2). GPS coordinates of sampling locations and topographic profiles were plotted in ArcMap^©^ using SPOT satellite imagery (2.5 m/pixel) (Fig. 1b) found in the ESRI World Basemap layer. Aerial photo imagery from LoftMyndir ehf (1.0 m/pixel) (Fig. 1c), WorldView-2 satellite (1.8 m/pixel), and UAV drone imagery were used to create a visual classification map delineating aʻa and pahoehoe surfaces into polygons based on the characteristics of pahoehoe having a smooth highly reflective surface and aʻa having a rough and weakly reflective surface (Byrnes et al. 2004; Crown and Ramsey 2017). Records of ground observations on topographic profiles and observations recorded during the eruption were also used in the creation of the visual classification map (Thorarinsson and Sigvaldason 1962; Thorarinsson 1963) (Fig. 2). Sentinel-2A L1C data were acquired by the sensor on August 2, 2019. ASTER L1B data were acquired by the sensor on August 7, 2012, both were retrieved from USGS Glovis (https://glovis.usgs.gov/) with cloud cover under 10%. All satellite datasets were projected in Transverse Mercator in UTM zone 27 on the WSG datum. Drone imagery was collected during the summer of 2019 with a Mavic 2 Pro Camera equipped with a 1' CMOS passive sensor. Estimated flow volume is calculated based on the detailed elevation data from topographic profiles (Additional file 1: Sheets 3–8) to estimate thicknesses of individual events across sections distributed along the entire flow length. The thickness is averaged for each event and is multiplied by the respective surface areas calculated in ArcMap (Table 2; Additional file 1: Sheet 9).

**Table 1:** A summary of analyses conducted on samples (denoted with a ‘x’)

Sample	Eruptive event	Distance from vent (km)	SEM crystallinity	MOSAIC vesicularity	Pycnometry bulk density	Pycnometry matrix density	Pycnometry connected porosity	ICP-MS Fe/Mg	EPMA olivine Fo#	EPMA plagioclase An#	EPMA pyoxene En#	EPMA glass Mg#
IIAU15-001	Vent Scoria	0.13		x	x	x	x	x				
IIAU15-002	Vent Scoria	0.13			x	x	x	x				
IIAU15-003	Vent Scoria	0.02			x	x	x	x				
IIAU15-004	Vent Scoria	0.00		x	x	x	x	x				
IIAU15-005	Vent Scoria	0.03		x	x	x	x	x				
IIAU15-006	Vent Scoria	0.02			x	x	x	x				
IIAU15-007	Vent Scoria	0.03			x	x	x	x				
IIAU15-008	Vent Scoria	0.08		x	x	x	x	x				
IIAU15-009	Pahoehoe 2	0.78		x	x	x	x	x				
IIAU15-011	Pahoehoe 2	1.30			x	x	x	x				
IIAU15-012	Pahoehoe 2	1.52	x	x	x	x	x	x	x	x	x	
IIAU15-013	A'a 2	2.07	x	x	x	x	x	x	x	x	x	
IIAU15-014	Pahoehoe 2	1.80		x	x	x	x	x				
IIAU15-015	Pahoehoe 3	3.94			x	x	x	x				
IIAU15-016	Pahoehoe 3	3.94		x	x	x	x	x				
IIAU15-017	Pahoehoe 2	4.03		x	x	x	x	x				
IIAU15-018	Pahoehoe 2	3.79		x	x	x	x	x				
IIAU15-019	A'a 1	5.28		x	x	x	x	x				
IIAU15-022	A'a 2	8.08	x	x	x	x	x	x	x	x	x	
IIAU15-023	A'a 2	9.52	x	x	x	x	x	x	x	x	x	x
IIAU18-001	A'a 1	3.38	x	x	x	x	x	x	x	x	x	x
IIAU18-002	A'a 2	3.50		x	x	x	x	x				
IIAU18-003	Vent Scoria	0.02		x	x	x	x	x				
IIAU18-004	Vent Scoria	0.05		x	x	x	x	x				
IIAU18-005	Vent Scoria	0.31		x	x	x	x	x				
IIAU18-006	Vent Scoria	0.16	x	x	x	x	x	x	x	x	x	x
IIAU18-007	Vent Scoria	1.52		x	x	x	x	x				
IIAU18-008	Vent Scoria	0.01		x	x	x	x	x				
IIAU18-009	Vent Scoria	0.09		x	x	x	x	x				
IIAU18-010	A'a 2	5.43		x	x	x	x	x				
IIAU19-001	Pahoehoe 3	3.51		x	x	x	x					
IIAU19-002	Pahoehoe 2	4.07		x	x	x	x					
IIAU19-003	Pahoehoe 3	0.45		x	x	x	x					
IIAU19-004	Pahoehoe 3	0.26	x	x					x	x	x	x
IIAU19-005	Pahoehoe 3	0.31		x	x	x	x					
IIAU19-006	A'a 2	8.93		x	x	x	x					
IIAU19-007	A'a 2	7.41		x	x	x	x					
IIAU19-008	Pahoehoe 3	3.80	x	x	x	x	x		x	x	x	
Vent Scoria	range		63%	15–46%	1.1–2.8 g/cm^3^	2.4–3.0 g/cm^3^	11–57%	1.97–2.2	60–67	59–68	41–59	7–34
A'a 1	range		85%	4–14%	2.8–3.0 g/cm^3^	2.9–3.0 g/cm^3^	0–1%	2.01–2.03	42–61	59–68	40–63	2–8
A'a 2	range		90–100%	2–36%	1.7–3.0 g/cm^3^	2.7–3.0 g/cm^3^	0–40%	1.95–2.01	30–59	46–70	41–65	5–7
Pahoehoe 2	range		86%	12–26%	2.6–3.0 g/cm^3^	2.6–3.0 g/cm^3^	0–8%	1.97–2.03	59–62	59–68	40–62	
Pahoehoe 3	range		55–79%	0–32%	2.6–3.0 g/cm^3^	2.5–3.0 g/cm^3^	0–13%	1.95–2.01	58–68	58–73	41–59	5–35
(n)			3	3	3	3	3	6	26	11	11	34
St. Dev			3.5%	1.5%	0.3 g/cm^3^	0.1 g/cm^3^	0.3 g/cm^3^	< 2%^a^	0.4	0.5	0.2	< 0.1

A summary of results are also given for each eruptive event in addition to associated uncertainties based off of repeated analyses (n) of a standard or methodology. See Additional file 1 for details

**Table 2:** Ranges of event flow thickness measured from Jacob’s Staff transects A-F (Figs. 1, 9) of eruptive events with calculated flow volumes using areal extent measured in ArcMap

Transect thickness	A	B	C	D	E	F	Avg. thickness of event flow (m)	Area (km^2^)	Volume (km^3^)
Event 1 A'a	2–8.5		3–8		0.3–3	3–26	6.4	5.67	0.036
Event 2 A'a	1–5		2.8–3.8	3–17	0.4–4	3–9	4.57	7.49	0.032
Event 2 Phh	0.25–1		0.6–0.9		0.2–0.5	1–4	0.85	1.62	0.001
Event 3	0.2–1	1. 5–4.5			0.1–0.5		0.99	0.07	0.000
Average transect thickness	7	1.5	5.3	9.6	4.2	14.6	7	Total volume	0.071

Backscatter images of eight samples were acquired from the Hitachi S-3400 scanning electron microscope at the University of Iowa (USA) to calculate total crystallinity (cumulative microphenocryst, and microlite abundance) and relative abundance of mineral phases with ImageJ (Additional files 2: Sheet 1–2, 4 and 5: Fig. S3). Cumulative phenocryst abundance was not calculated as phenocrysts are rare. Thin section scans were produced from 33 rock samples and processed with Modeling Object Structure and Analysis Information Calculator, a software application designed to produce quantitative morphometrics and model structures for geological digital images (Additional file 2: Sheet 3). Bulk and matrix densities and connected porosity were measured for 38 rock samples at the University of Auckland, New Zealand, using a micrometrics Geopyc 1360 and Accupyc 1340 (Additional file 2: Sheet 4). Lava flow viscosity was estimated based on density (ρ, kg/m^3^), flow velocity (v, m/s), base slope (α, °), and flow thickness (h, m); η = ((ρgsinα)/2v)) *h^2^ (Tallarico and Dragoni 1999, Eq. 23), where η is relative viscosity (Pa·s) and g is the gravitational constant 9.81 m/s^2^. Flow velocity relies on recorded observations of the average advancement rates for flow fronts (Fig. 2 and Additional file 2: Sheet 5).

Whole-rock major and trace element data were measured with a Thermo X-series Inductively Coupled Plasma Mass Spectrometer (ICP-MS) at the University of Iowa (USA) following the methods of Peate et al. (2010) and Reagan et al. (2013) for all rock samples collected in 2015 and 2018. The other samples collected in 2019 were analyzed using a 193 nm excimer laser ablation ICP-MS for major element and a Thermo Scientific iCAPQ ICP-MS for trace element concentrations at Michigan State University (Additional file 3: Sheet 1). A total of eight samples from the eruption were analyzed with a JEOL JXA-8230 Electron Microprobe Analyzer (EMPA) at the University of Iowa (USA) for mineral phase compositions of olivine, plagioclase, pyroxene, and glass (Additional file 3: Sheets 2–5). The plagioclase-liquid geothermometers from Putirka (2005) (also referenced in Putirka 2008: Eq. 23) and Putirka (2008: Eq. 26) were used to calculate plagioclase crystallization temperature at 1 atm. If the difference in temperatures between the two geothermometers is within ± 48 °C, then the crystal-liquid pairs are considered to be in equilibrium (Additional file 3: Sheet 6). The liquid-only geothermometer was also used (Putirka 2008: Eq. 14) in addition to the cpx-liquid geothermometer (Putirka 2008: Eq. 33). Water was assumed to be 0.5 wt.% after Nichols et al. (2002) for all thermometry calculations (Additional file 3: Sheet 6).

Detailed methodology descriptions are available in Additional file 4 and a summary of analyses conducted on samples with associated uncertainties is in Table 1.

## Results

### Reinterpreting the eruption sequence from the surface textures of lava flows

This study integrates the first-person observations of the eruption (Thorarinsson and Sigvaldason 1962; Thorarinsson 1963), with modern satellite imagery, aerial photos, and new field observations to provide an updated and more detailed description of the eruption progression.

Morphotypes are distinct surface morphologies of a lava flow that reflect both internal parameters and external influences (Murcia et al. 2014). We refer to these characteristics as texture, which encompasses the correlations among large-scale surface morphology, intermediate-scale ground-based observations of lava structures, and microscope-scale petrographic fabrics. We have divided the eruption into both spatial and temporal sequences based on similarities in eruption and emplacement mechanisms and resultant physical characteristics.

Additionally, we utilize the following lava structure terms: open aʻa channel as defined by Lipman and Banks (1987) who observed channelized aʻa flows in Mauna Loa; levee types defined by Sparks et al. (1976); and basaltic texture types including shelly pahoehoe, slabby pahoehoe, spiny pahoehoe, scoriaceous aʻa, clinker aʻa, and blocky aʻa as described by Swanson (1973), Peterson and Tilling (1980), and Lipman and Banks (1987). We utilize the term “lava flow” as the entire moving lava body during a given eruptive event. Flow lobe refers to specific lava flow branches after bifurcation, as exhibited in Fig. 1.

### Event 1 aʻa emplacement

**Fig. 3:**
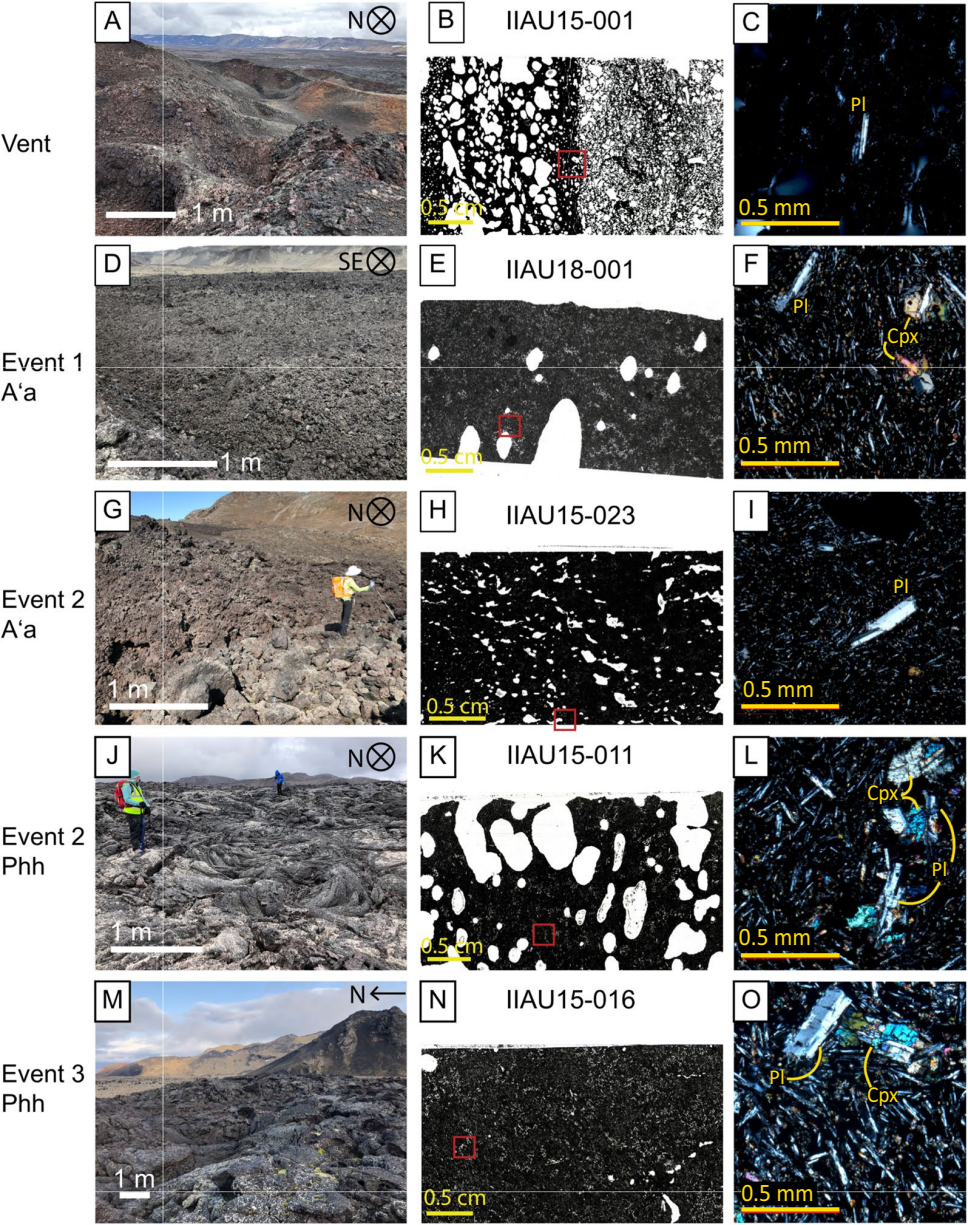
Representative textures of 1961 lava flow; white scale bar in field images is 1 m; yellow scale bar in thin section scans is 0.5 cm, red boxes indicate locations of photomicrographs; red scale bar for photomicrographs (4 × magnification) is 0.5 mm. ‘pl’ = plagioclase; ‘cpx’ = clinopyroxene. **A** Vent scoria textures from E vent facing N with oxidized spatter and scoria. **B** Thin section scan of IIAU15-001, from the S face of E vent, exhibits both end-member vent textures. **C** Photomicrograph of IIAU15-001 showing a single plagioclase microphenocryst surrounded by a glassy microlite-rich groundmass. **D** Clinkery and blocky event 1 aʻa on transect F at the S lobe, facing SE. **E** Thin section scan of IIAU18-001, taken from the bottom of an aʻa levee at the end of transect A. **F** Photomicrograph of IIAU18-001 containing a microphenocryst of plagioclase and microphenocrysts of pyroxene. **G** Blocky event 2 aʻa taken at start of the N lobe at transect E near branching point 2 (I.A. Ukstins for scale). **H** Thin section scan of IIAU15-023, sampled from the flow front of the middle lobe. **I** Photomicrograph of IIAU15-023 with a plagioclase microphenocryst surrounded by a groundmass of plagioclase, pyroxene, olivine microlites, and glass. **J** Shelly and ropey event 2 pahoehoe photographed on the middle lobe at transect A facing N. **K** Thin section scan of IIAU15-011 sampled 1 km from the vents. **L** Photomicrograph of IIAU15-011 with microphenocrysts of pyroxene and glomerocrysts of pyroxene and plagioclase. **M** Shelly pahoehoe from the 3rd event on the W halo finger photographed at transect B. **N** Thin section scan of sample IIAU15-016, from the lava flow interior of the W halo finger. **O** Photomicrograph of IIAU15-016 with a large glomerocryst of plagioclase and pyroxene

The eruption initiated with aʻa lava—event 1 aʻa flows are from 1.5 to 16 m thick, range from 0.25 to 1.1 km wide and have clinker surface breccia with clasts 10 cm–3 m in diameter forming a rubbly upper carapace (Fig. 3d). Aʻa clasts are randomly arranged with sharp edges due to broken vesicles at the surface and appear black but may have dark red patches from oxidation (Fig. 3d). Among the clasts are armored lava balls (as defined by Stearns 1926 and Wentworth and MacDonald 1953) which are the result of lava clasts rolling along the flow surface accumulating molten material like a snowball. The lava balls reach diameters of 4 m with cores of layered, highly scoriaceous oxidized clasts that resemble lithified vent deposits. At 4 km downflow, event 1 aʻa bifurcated into two lobes upon interaction with a topographic high and formed a kipuka (Fig. 1c) creating a “north lobe” and “south lobe” (Fig. 1c). From the branching point (labeled on Fig. 1 as branch 2), the north lobe flowed 0.25 km; the southern lobe flowed 2.6 km, where it bent south and continued for another 1.4 km for a total downflow distance of 8 km (Fig. 1c).

### Event 2 aʻa emplacement

**Fig. 4.**
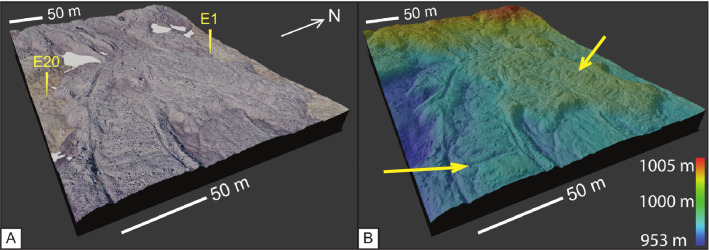
3-D rendered image from UAV DEM data at the initiation of the north lobe, which aligns with the start of transect E (Fig. 1c). The image is looking up-flow to accentuate the festoon texture seen in the foreground, which is identified with the black arrow. The background displays the rubble levees of event 2 aʻa flows. Numbers after transect label E refer to GPS locations (see Additional file 1: Sheet 7)

The second stage of the eruption started with aʻa flows ranging from 3 to 18 m in thickness and 0.25 to 1 km in width and becomes exposed directly east of the vents. At 1.6 km, event 2 aʻa displays distinct, large-scale surface ridge textures that are resolvable at horizontal resolutions of 10 m/pixel (Fig. 1c and Fig. 4). These ridges are identified here for the first time as festoons, which are defined as a subtype of pressure ridge found on pahoehoe flows at the centimeter scale (Wentworth and MacDonald 1953; Larson 1991), on flood basalts at the meter scale (Theilig and Greeley 1986), on andesites and dacites at the meter scale (Oizumi et al. 2018), on rhyolites at the meter scale (Fink 1980), and on extra-terrestrial flows at the meter to kilometer scale (Theilig and Greeley 1987; Wroblewski et al. 2019). Festoons on event 2 aʻa flows are 5 m high, 10 m from crest to crest, and span the entire 0.2 km width of the lobe, which is bounded by rubble levees. These festoons are reminiscent of pahoehoe ropes, but occur on a much larger scale and are composed entirely of brecciated aʻa ranging from 10 cm – 3 m in diameter (Fig. 3g).

The event 2 aʻa lobe with festoon texture bifurcated twice and festoons are present for 2.8 km after branching point 1 on lobe 1 (Fig. 1c). The festoon texture in lobe 1 is disrupted after flowing down a stepped surface with about 10 m of drop over a horizontal distance of 70 m, at which point the aʻa surface becomes hummocky (Fig. 1c).

In the north lobe (Fig. 4) after branch 2 (Fig. 1c), event 2 aʻa was emplaced onto, and covered, event 1 aʻa where it flowed in a single channel surrounded by its own rubble levees for 2 km. The aʻa channel also exhibits a smaller scale festoon texture (40 m long) (Fig. 4). The levees in the north lobe became more pronounced as the aʻa flowed down a ~ 10 m step, after which it transitioned to dispersed flow for the final 1 km length, reaching a total distance of 7.4 km at a width of 0.5 km and a thickness of about 5 m. The southern lobe of event 2 aʻa also displays festoons that are about 100 m wide and 1 m tall, with a crest wavelength of 15 m.

The middle lobe of event 2 aʻa was the final stage of this sequence and was emplaced over the southern event 2 aʻa lobe. The middle lobe has distinctive accreted levees with brecciated talus slopes and near vertical inner walls up to 5 m high. The middle lobe transitions to weakly channelized flow with marginal shear ridges for 2 km and then transitions to dispersed flow for 1.5 km, where it reached the maximum lava flow extent of 9 km with an average thickness of 5 m at the flow front (Fig. 1c). Sample IIAU18-10 was collected in the middle lobe of the event 2 aʻa. It is from a rafted scoria cone chunk (or lava boats: Lipman and Banks 1987) which appear as 2–4 m conical vent edifices or as large sub-spherical lithified boulders resting on the aʻa surface. We observed that the rafted scoria cone chunks are covered by vesicle-rich spiny aʻa and have vertical-to-angled (45˚) frictional striations, with 0.5 cm spacing, from scraping of ductile lava.

### Event 2 pahoehoe emplacement

The final stage of event 2 emplaced shelly pahoehoe, which erupted at the vents and flowed to the east 1.3 km with a width of about 0.5 km (Fig. 1c). Pahoehoe from event 2 is also found at distances greater than 1.3 km and emerges as breakouts from event 2 aʻa. Pahoehoe has slabby, spiny, and shelly textures (Fig. 3j), with collapsed skylights. Pahoehoe breakout 1 (‘PB1’ in Fig. 1c) transitions into aʻa lava, which then traveled an additional 0.5 km east where the final pahoehoe breakout emerged from it (‘PB2’ in Fig. 1c).

### Event 3 pahoehoe emplacement

Eruptive event 3 initiated with the emplacement of ropey to shelly pahoehoe (Fig. 3m) which emerged on the western side of the vent area, forming a pahoehoe halo about 100 m wide (Fig. 1c). Event 3 pahoehoe also emerged from tubes 1.5 km east of the vents on the southern margin of the 1961 lava and flowed east for 4 km, confined by older lavas to the north and the caldera wall to the south.

### Flow volume estimates

The total flow volume estimated in this study is about 0.07 km^3^, which matches closely with the original volume estimation of 0.1 km^3^ (Thorarinsson and Sigvaldason 1962; Thorarinsson 1963). Our detailed observations validate the original volume estimates and provide additional detail into the flow volume for each eruptive event. Event 1 emplaced ~ 0.04 km^3^ of aʻa lava, event 2 emplaced ~ 0.03 km^3^ of aʻa lava and ~ 0.001 km^3^ of pahoehoe lava, and finally event 3 emplaced ~ 0.0001 km^3^ of pahoehoe lava (Table 2).

## Internal textures

### Petrographic descriptions

**Fig. 5:**
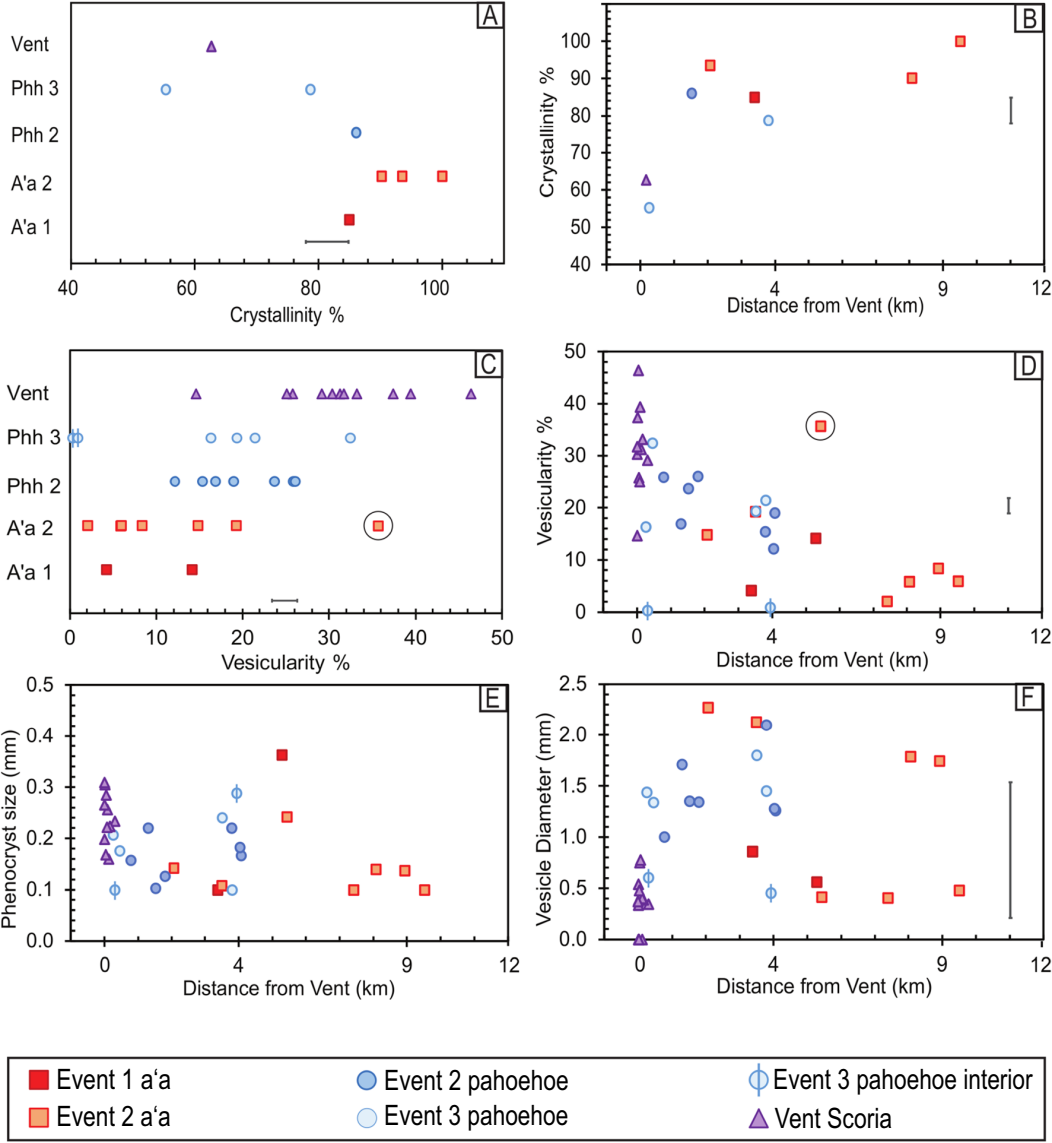
**A**–**D** Plots of total crystallinity (%) and vesicle abundance (%) showing the range and distribution across textural types and variations with distance from the vents, using SEM ImageJ and MOSAIC* analyses. Error bars are ± 1 s.d. **E** Weighted average phenocryst sizes (plagioclase + pyroxene) and **F** vesicle sizes both do not show any apparent trends with distance from the vents or between the texture types. Anomalous sample IIAU18-010 is circled (see text)

All lava samples contain plagioclase and pyroxene phenocrysts (> 0.5 mm) and microphenocrysts (0.5–0.05 mm) surrounded by a microlite-rich groundmass (< 0.05 mm). Plagioclase is the most abundant mineral phase (~ 1–20% modal abundance) and consists mainly of thin individual lathes (0.2 mm) or radial glomerocrysts (< 1 mm) ± pyroxene (Fig. 3). Large phenocrysts of plagioclase (> 1 mm) are present but rare and are typically euhedral to subhedral with sieve textures. No evidence of melt inclusions have yet been observed in thin sections. Pyroxene are euhedral to subhedral with modal abundance from 0.5 to 10%. Pyroxene are generally small (< 0.2 mm) (Fig. 3) but can be up to 1 mm in size. Rare olivine microphenocrysts (< 1%) are small (0.15 mm), but in the groundmass have an average modal abundance of 9% ranging from 0 to 20%. Groundmass olivines are typically < 0.05 mm, as measured on SEM backscatter images (Additional file 5: Fig. S3). Granular and sometimes skeletal (~ 0.03 mm) Fe-Ti oxides are present in the groundmass at modal abundances up to 6%. Glass is found in the surface rinds from flowtops as well as interstitially in the groundmass and ranges from 0 to 50% by volume (Fig. 5a and Additional file 5: Fig. S3). Crystallinity is dominated by microphenocrysts and microlites, as phenocrysts are relatively uncommon.

## Vent scoria: vesicularity and crystallinity

**Fig. 6:**
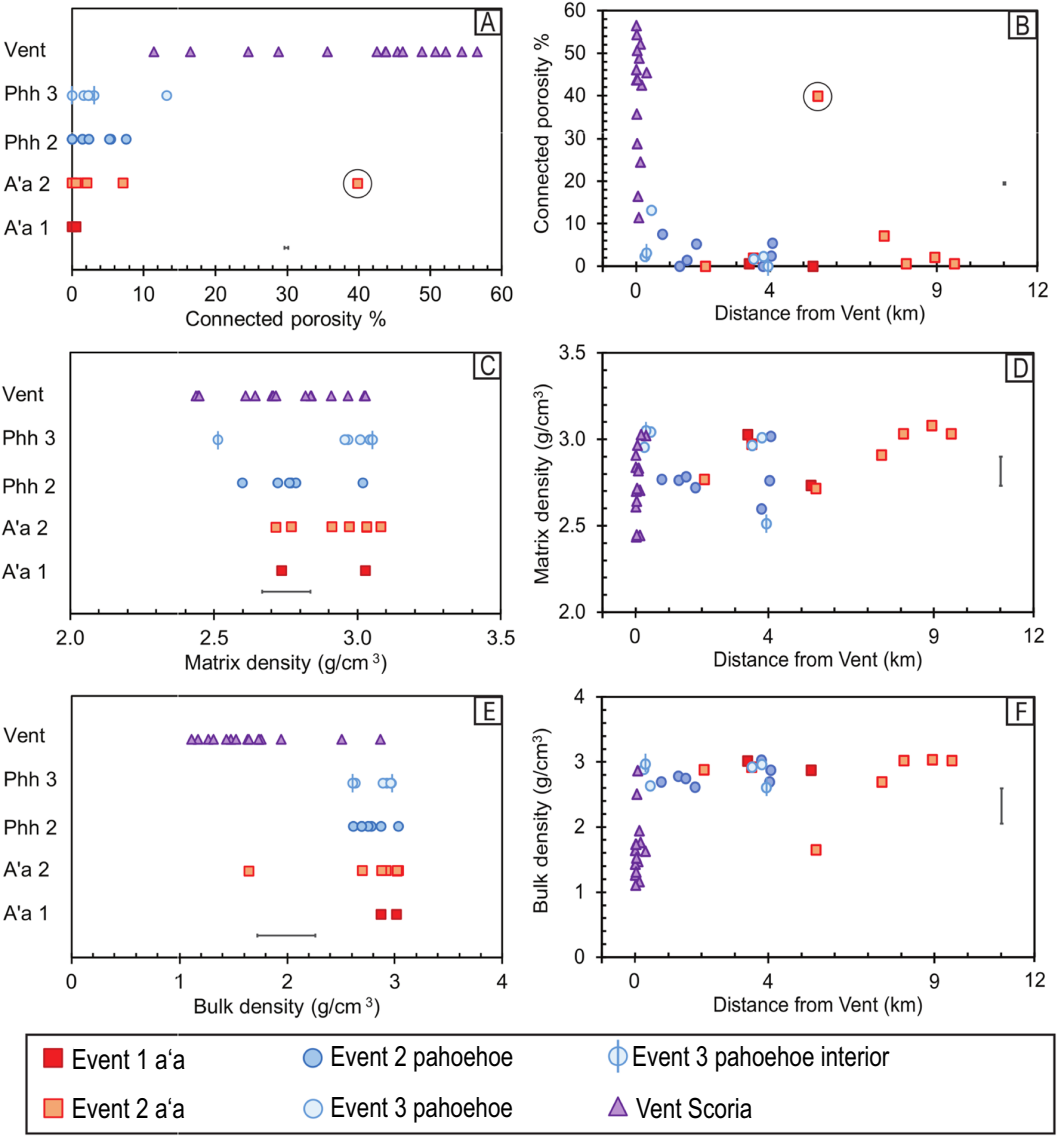
**A**–**F** Variability in matrix density, bulk density, and connected porosity with respect to event texture and distance from the vents. Error bars are ± 1 s.d. see Additional file 2: Sheet 4 for further details. Anomalous sample IIAU18-010 is circled (see text)

Total crystallinity of the vent scoria averages 63% and shows two textural endmembers. The most common texture is small (0.1–0.2 mm) high-abundance microphenocrysts of plagioclase and pyroxene (5–10% total) (Fig. 3) in a glass-rich, highly vesicular groundmass (~ 32% by area and small vesicles about 0.5 mm in diameter) (Fig. 5a). The other end-member texture in vent samples is low-abundance microphenocrysts of plagioclase and pyroxene (1–2% by volume, 0.2 mm in length) (Fig. 3) with irregularly shaped larger sized vesicles (~ 25% by area with sizes ranging up to 0.7 mm) (Fig. 5a). Sample IAU15-001 (Fig. 3b, c) exhibits bands of both textures. Vent scoria have high connected porosity of 57% (Fig. 6a) corresponding to high amounts of vesicle coalescence (Fig. 3b). Densities range from 2.0 to 3.0 g/cm^3^ (Table 1; Fig. 6c and e).

Samples from the southern-most and youngest vent (Fig. 2) (IIAU18-003 and IIAU18-004) have the highest abundance of microphenocrysts—10–20%—compared to other vent samples which generally have a maximum of 9.5%. The youngest vent rocks have vesicle abundances of 37–25%, respectively (Fig. 5a), with oblong shapes and average sizes of 0.5–0.7 mm.

### Event 1 aʻa: vesicularity and crystallinity

Total crystallinity averages 85% (Table 1; Fig. 5a). Plagioclase is the most abundant mineral (~ 2–15% modal abundance) and has an average size range of 0.1–0.4 mm (Fig. 3). Pyroxenes are slightly smaller from 0.1 to 0.2 mm with modal abundances of 1–5%. All microphenocrysts are surrounded by a microlite-rich groundmass (plagioclase: ~ 47%, pyroxene: ~ 31%, olivine: ~ 4%, oxides: ~ 3%, and glass: ~ 15%) (Fig. 3). Olivines are small (< 0.05 mm) and subhedral. Fe–Ti oxides are small (< 0.05 mm) granular-to-skeletal grains. Vesicles are about 4–14% abundant (Fig. 5c) with sub-circular or elongated shapes that range from 0.6 to 1.0 mm in diameter (Fig. 3). Samples have low connected porosity of 0–0.6% (Fig. 6a) with densities of 2.8 g/cm^3^ (Table 1; Fig. 6c).

### Event 2 aʻa: vesicularity and crystallinity

Event 2 aʻa crystallinity ranges from 90 to 100% (Table 1; Fig. 5a). Plagioclase microphenocrysts are 0.1–0.16 mm with low modal abundances of about 1–5%. Pyroxene microphenocrysts are 0.1–0.12 mm with modal abundances up to 3%. The groundmass is microlite-rich, composed of plagioclase (40–52%), pyroxene (32–42%), minor olivine (0–14%), oxides (5%), and glass (0–8%). Groundmass olivine are small (< 0.05 mm) and subhedral to euhedral. Oxides are skeletal or granular and are up to 0.05 mm. Vesicles are 2–36% abundant (Table 1) ranging in size from 0.4 to 2 mm, where total vesicularity increases with increasing vesicle size (Fig. 5c). The connected porosity is up to 7% with an average of about 2% (Fig. 6a). Matrix and bulk density are the same, at 2.9 g/cm^3^ (Table 1; Fig. 6c and e).

Sample IIAU18-010, a rafted scoria cone chunk sample, has high vesicularity (35%) and high connected porosity (40%) and is inferred to be a rafted block of vent material.

### Event 2 pahoehoe: vesicularity and crystallinity

Total crystallinity averages 86% (Table 1; Fig. 5a). Plagioclase microphenocrysts range from 5 to 15% (0.1–0.2 mm in size) while pyroxene microphenocrysts are 1–7% abundant (0.1 mm) (Fig. 3). The groundmass is composed of microcrystalline plagioclase: ~ 38%, pyroxene: ~ 25%, olivine: ~ 19%, oxides: ~ 4%, and glass: ~ 14%. Olivine crystals are euhedral to subhedral and are typically < 0.05 mm, but a rare olivine microphenocryst (0.15 mm) is present. Oxides are skeletal to granular and are < 0.01 mm in size. Vesicles are 1.0–2.0 mm in diameter and have abundances from 12 to 26% (Table 1 and Fig. 3c). Vesicles under 1.6 mm are more spherical, and larger vesicles are typically more elongated (Fig. 3). Connected porosity ranges from 0 to 8% (Fig. 6a) and bulk density is 2.7 ± 0.1 g/cm^3^ (Table 1; Fig. 6c and e).

### Event 3 pahoehoe: vesicularity and crystallinity

Total crystallinity is 55–79% (Table 1; Fig. 5a). Plagioclase microphenocrysts have abundances of 7–20% and are 0.1–0.3 mm in length. Pyroxenes are 3–7% abundant and are about 0.1 mm in size (Fig. 3). The groundmass contains plagioclase: 16–32%, pyroxene: 31–36%, olivine: 7–10%, oxides: 0–4%, and glass: 19–46%. Groundmass olivine are euhedral to subhedral and are no larger than 0.05 mm. Olivine may be present as microphenocrysts, but they are very low (< 1%) in modal abundance and are 0.1 mm in size. Oxides in the groundmass are mostly skeletal and about 0.01 mm in size. Vesicles in pahoehoe are elongate and range in size from 1.3 to 1.6 mm with abundances from 17 to 36% (Table 1; Fig. 5c and f). Connected porosity is very low, ranging from 0 to 13% (Fig. 6a). Bulk and matrix density are both 2.8 ± 0.3 g/cm^3^ (Table 1; Fig. 6c and e).

### Down-flow variation of crystallinity and vesicularity

Crystallinity values are distinct between eruption textures and reflect the abundances of microphenocrysts and microlites. Event 1 aʻa samples have high crystallinity of > 85% which increases downflow at 1% per km. Pahoehoe samples have lower values from 55 to 86%, but increases at higher rates of 5% per km (Fig. 5b).

Vesicle abundance is strongly related to distance from the vents (Fig. 5d) (Polacci et al. 1999; Robert et al. 2014). Vesicularity is similar between pahoehoe and aʻa lavas located the same distance from the vents—at downflow distances of 2–4 km all lavas have vesicularity of 12–20% (Fig. 5d). It decreases by about 2% per km irrespective of lava type or transport mechanism, where pahoehoe lavas were transported for the first few kilometers from the vents via lava tubes while aʻa flows were emplaced largely at the surface for the entire distance.

### Viscosity

Calculations based on the model from Tallarico and Dragoni (1999) yield event 1 aʻa viscosity ranges from 10^3^ to 10^4^ Pa·s. Event 2 aʻa has a range of 10^4.7^ – 10^5.6^ Pa·s, event 2 pahoehoe ranges from 10^2.3^ to 10^3.7^ Pa·s, and event 3 pahoehoe ranges from 10^2.7^ to 10^3.6^ Pa·s. Calculations may be viewed in Additional file 2: Sheet 5.

### Whole-rock chemistry

**Fig. 7:**
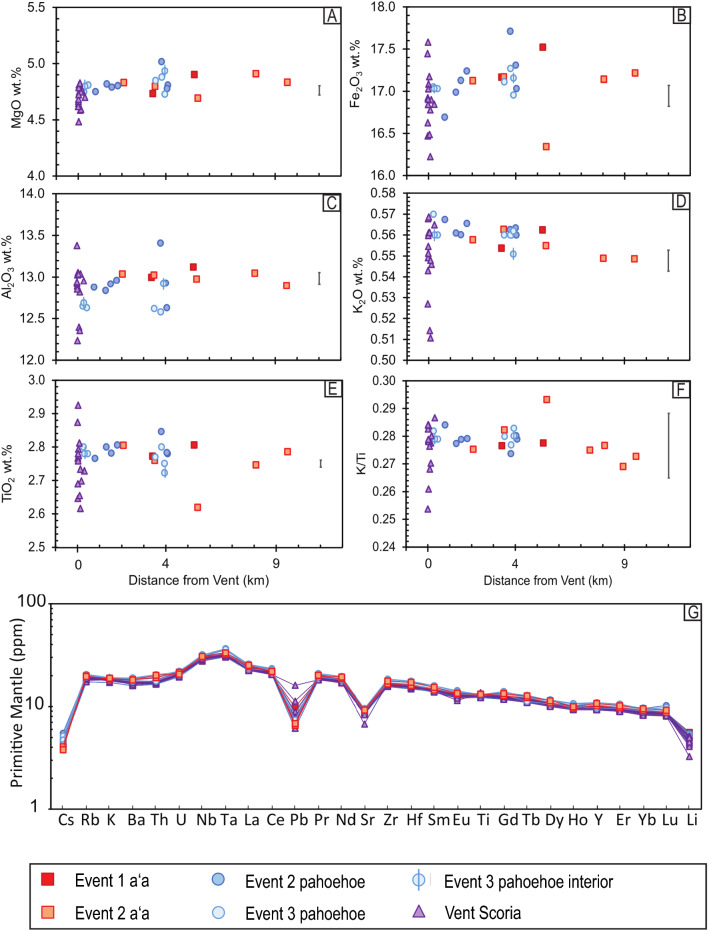
**A**–**F** Whole-rock major element composition with distance from the vents to the flow front. The error bar is ± 1 s.d. measured with the reproducibility of the W-2 standard—see Additional file 3: Sheet 1 for further details. **G** Primitive-mantle normalized trace element patterns (normalizing values: Sun and McDonough 1989)

The 1961 eruption has an evolved tholeiitic basalt composition, with MgO from 4.5 to 4.9 wt.%, SiO_2_ from 47 – 52 wt.%, FeO from 16 to 18 wt.%, Na_2_O from 2.2 to 2.9 wt.%, K_2_O from 0.51 to 0.57 wt.%, and low Ni from 21 to 32 ppm (Fig. 7). The Fe/Mg ratio is 1.9–2.2.

The compositional homogeneity of the 1961 flow is also seen in the trace element data. All samples have virtually identical primitive-mantle-normalized trace element patterns (normalizing values: Sun and McDonough 1989) (Fig. 7g), and ratios of incompatible elements show limited variations, such as K/Ti (0.28 ± 0.01: Fig. 7f), La/Sm (2.50 ± 0.02), and Nb/Zr (0.114 ± 0.002). Vent scoria samples show more variations in Pb (1.1 to 4.5 ppm) compared to lava samples (1.2 to 1.8 ppm), which is likely related to eruptive degassing of volatile Pb and formation of Pb-rich condensates in the vent material (e.g., Aiuppa et al. 2003). A negative Sr anomaly and Eu/Eu* < 1 (0.92 ± 0.01) indicate plagioclase fractionation (Fig. 7g, Additional file 3: Sheet 1).

### Mineral and glass chemistry

**Fig. 8:**
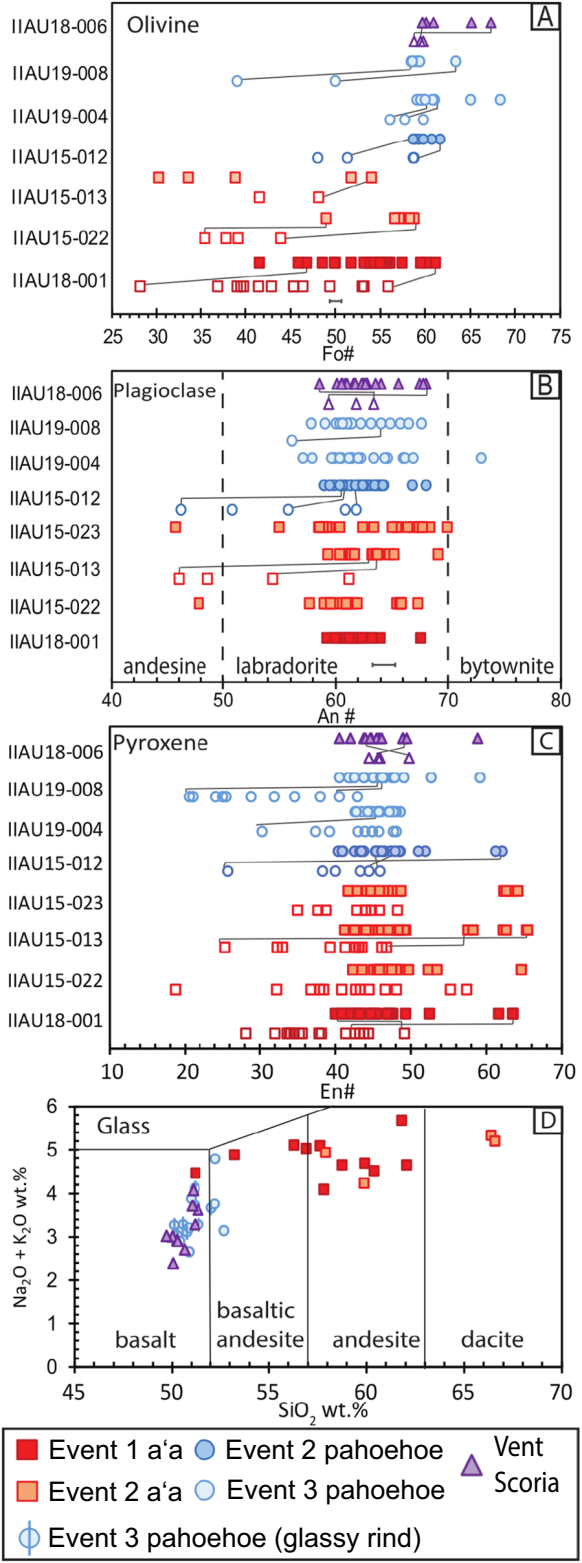
Mineral and glass compositions are plotted temporally—with respect to event and lava texture. Open symbols denote rim analyses and colored symbols denote core analyses. The grey lines connect some core and rim pairs from the same crystal. Error bars are 1 s.d.% based on replicate standard analyses: see Additional file 3: Sheets 2–5. **A** Olivine compositions are presented with Fo# (molar Mg/[Mg + Fe^2+^]). **B** Plagioclase compositions are presented with An# (molar Ca/[Ca + Na + K]). **C** Clinopyroxene results are presented with En # (molar Mg/[Mg + Fe^Total^]). **D** Glass compositions are plotted on a TAS diagram and show the difference between surface and interstitial glass. Pahoehoe and vent samples measured mostly surface glass with minor interstitial glass and are basaltic

Olivine microlites have a calculated Fo# (molar Mg/[Mg + Fe]) range of 28–68 (Table 1). Olivines from the vents have the least compositional variability (Fo# 60–67) and are mostly unzoned. When zonation is present, the rims consistently have lower Fo# (58.7–59.7) than their respective cores with differences in Fo# from 9 to 13 (Fig. 8a). The olivine cores of vent samples have a similar compositional range of Fo# 60–67 to those from late stage pahoehoe lavas of event 2 and 3 (Fo# 58–68: Fig. 8a). Olivine cores from aʻa in events 1 and 2 have the lowest recorded Fo# of 30–60 with the lowest Fo# of 28 in event 1 aʻa (Fig. 8a). Olivine rims from pahoehoe flows span a slightly more primitive Fo# range of 39–60 and are more evolved than their respective cores (Fig. 8a). Other chemical components in olivine, such as CaO, NiO, and TiO_2_ have ranges that are less than 0.5 wt.%. However, chemical zoning with respect to CaO and MnO is present, where cores are depleted relative to their crystal rims in the lava flow but are relatively enriched compared to the rims at the vents (Additional file 3: Sheet 2).

Plagioclase microlites have An# range of 46–73 (An# = molar Ca/[Ca + Na + K]) (Table 1). While the overall ranges of plagioclase vary slightly between event flows, the average is constant at an An# of 62 (Fig. 8b). The vents have the smallest observed range (An# 58–68) and are identical to the crystals from event 1 aʻa (Fig. 8b). Event 2 aʻa have the largest compositional range in An# of 46–70 and contain the most andesine, with two in event 2 aʻa crystal cores and two more in event 2 aʻa crystal rims (Fig. 8b). The only other sample with andesine is event 2 pahoehoe, which also has a large compositional range of An# 46–68. Event 3 pahoehoe (An# 57–73), contains the only analyzed bytownite crystal (An# 73) (Fig. 8b; Additional file 3: Sheet 3).

Clinopyroxene microlites are mainly augitic with minor amounts of pigeonite (Table 1). All pyroxene cores are 40–65 En# (molar Mg/[Mg + Ca + Fe]). All crystal rims have En# 20–48 and are typically more evolved than their respective cores (Fig. 8c). There are 5 analyzed pyroxene rims with higher En# than their respective cores. Pyroxene cores show 2 compositional groups, a dominant cluster of augites between En_42_Fe_20_Wo_38_ and En_52_Fs_23_Wo_25_ and a minor cluster of pigeonite between En_62_Fe_26_Wo_12_ and En_63_Fe_26_Wo_11._ Titanium (TiO_2_) in all pyroxene samples ranges from 0.3 to 3.8 wt.% and shows the largest enrichment in crystal rims from pahoehoe (1.5–3.8 wt.%). Manganese (MnO) in pyroxene has a small range of 0.23–0.66 wt.% through the entire eruption and is typically enriched in the rims relative to the crystal cores (Additional file 3: Sheet 4).

Two types of glass were analyzed: glass located at the lava surface (in vent and event 3 pahoehoe samples) and interstitial glass located between crystal grains in the groundmass of samples (in event 1 and 2 aʻa). Glass from the vent samples and event 3 pahoehoe have similar compositional ranges, predominately containing basaltic compositions (50–52 wt.% SiO_2_) (Fig. 8d). All vent and pahoehoe glass are compositionally distinct from the more evolved interstitial glass analyzed in aʻa samples. There is large overlap in glass compositions between event 1 and event 2 aʻa flows, but event 1 has a lower range in SiO_2_ (51–63 wt.%) than event 2 (> 66.4 wt.% SiO_2_) (Fig. 8d). Alkalis (Na_2_O and K_2_O) in glass are similar, however vent scoria and pahoehoe flows are lower than aʻa (2.7–4.8 vs 4.0–5.7 wt.% Na_2_O + K_2_O; Fig. 8d; Additional file 3: Sheet 5).

## Discussion

### Differences in eruption sequence interpretation

The eruption progression of the 1961 lava as interpreted from field and satellite observations matches the official eruption reports, which were conducted at the eruption site and provide continuous ground documentation of effusion rate, flow advancement rate, vent activity, and flow lobe emplacement from Oct. 26–Nov. 7 (Fig. 2) (Thorarinsson and Sigvaldason 1962; Thorarinsson 1963). After Nov. 7, observations were recorded exclusively by airplane due to harsh weather conditions limiting observations. It is also in this time frame, during event 3 pahoehoe emplacement (Nov. 27–28), where our interpretation differs from the reports. It is stated that event 3 pahoehoe emerged from tubes 7 km downslope from the vents, but satellite imagery shows no evidence of pahoehoe at that location. It is possible that this observation was referring to the 2nd pahoehoe breakout (PB2 in Fig. 1) which starts 4 km downslope from the vents and extends to ~ 5.5 km, but PB2 breakout is interpreted to be from the second eruptive event.

### Differences in eruptive event viscosity calculations

**Fig. 9:**
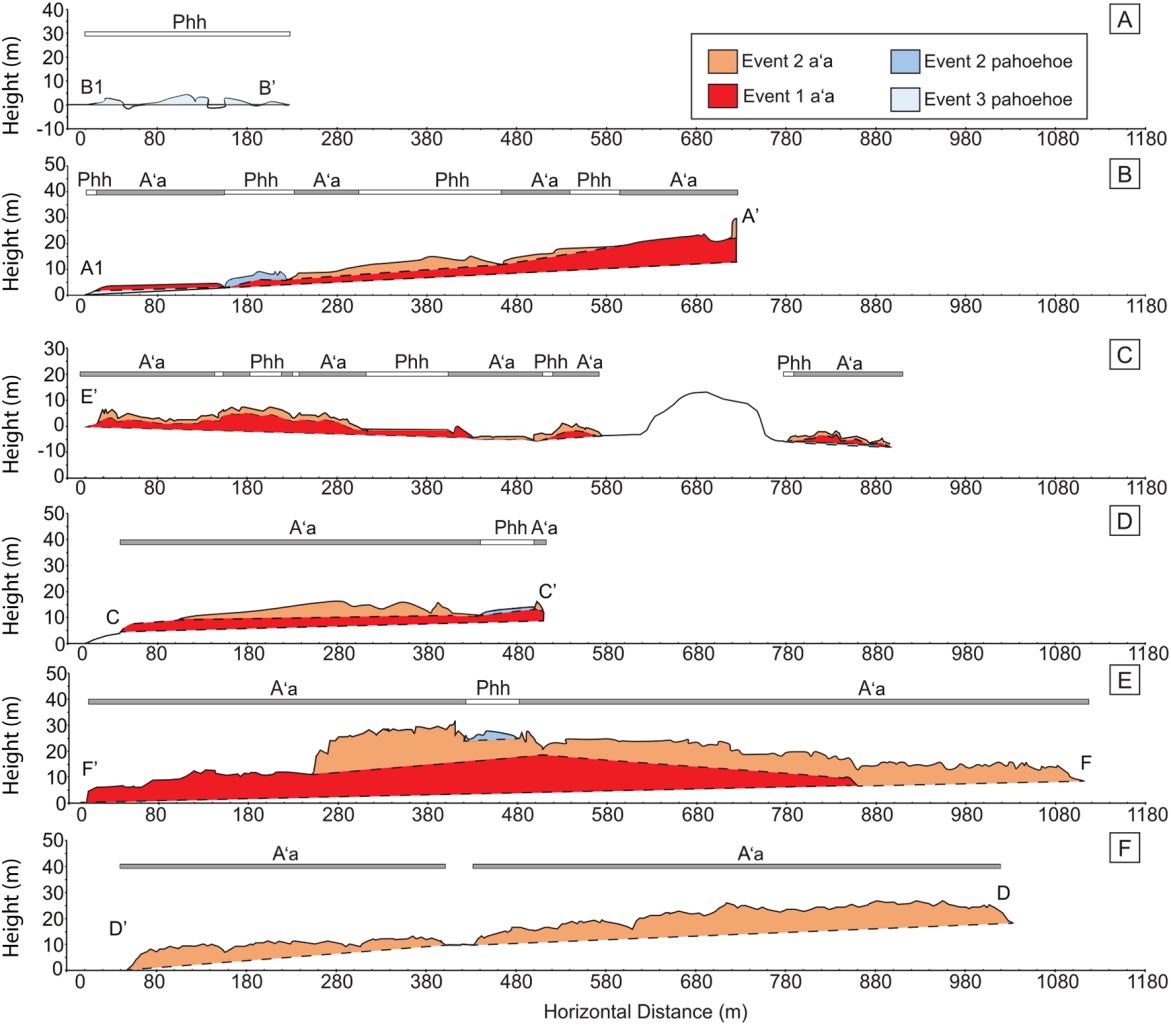
**A**–**F** Topographic profiles are plotted with 4 × vertical exaggeration (see Fig. 1c for locations). All profiles are oriented from the southern edge of the lava flow to the northern edge and arranged from closest to the vents to farthest from the vents. Dashed lines correspond to interpolated or estimated boundaries

Temperature is a main controlling factor of lava viscosity in a system that is otherwise compositionally or texturally uniform (Sehlke et al. 2014). Despite the independence of Tallarico and Dragoni’s (1999) viscosity model to temperature, the simplification of it creates a large dependence towards the observed flow velocity instead. The influence of temperature to the viscosity estimation is naturally incorporated into the flow velocity, as neither composition nor density is changing across eruptive events. The large viscosity range of event 2 pahoehoe is attributed to the difference in flow speed of surface flows and breakout flows (Thorarinsson 1963). Surface flows have the lowest calculated viscosity of 10^2.3^ Pa·s while the tube breakout flows, which were much slower to emplace (Thorarinsson 1963), have a higher calculated viscosity of 10^3.7^ Pa·s. The general similarity between calculated viscosity values of aʻa and pahoehoe indicate that the viscosity variations within these are likely dependent on factors outside of density, channel morphology, or underlying topography, which are relatively consistent across the entire eruption (Figs. 6, 9).

### Whole-rock chemistry influence on lava texture

The whole-rock compositions of the 1961 eruption are homogenous and indistinguishable (Table 1, Fig. 7a and b), as exemplified by the constant MgO concentrations (4.8 ± 0.1 wt.%) across all units (Fig. 7a) and the virtually identical primitive-mantle normalized trace element patterns (Fig. 7g). The general compositional similarities between vent, aʻa, and pahoehoe samples suggest that chemical composition is not the dominant control on observed lava texture. Additionally, the homogeneity of MgO indicates that all flows experienced similar extents of fractionation from the same magma batch. Geochemistry data are consistent with previously published compositions (Thorarinsson and Sigvaldason 1962; Kuritani et al. 2011) (see Additional files 3: Sheet 7 and 5: Fig. S1) and are compositionally similar to the evolved twentieth century basalts of the Askja volcanic system (Hartley and Thordarson 2013).

### Mineral–melt equilibrium

Mineral chemistry is related to lava viscosity due to trends in equilibrium crystallization that occur during cooling and subsequent thickening (Sehlke et al. 2014; Soldati et al. 2018). The presence of highly evolved olivine microlites and more primitive (enstatite and ferrosilite rich) pyroxene microphenocryst and microlite rims relative to their crystal cores are worth further consideration.

Mineral–melt equilibrium of olivine, pyroxene, and plagioclase are constrained following the tests of Roeder and Emslie (1970), Putirka (2008), Neave and Putirka (2017), and Neave and Namur (2022). The Rhodes diagram (Roeder and Emslie 1970) plots Mg# of the liquid vs. Fo# (or Mg#) of the crystal with respect to an equilibrium field determined by the partition coefficient K_d_(Fe–Mg) = 0.3 ± 0.03 for olivine, K_d_(Fe–Mg) = 0.27 ± 0.03 for pyroxene, and K_d_(Ca–Na) = 1.15 ± 0.15 for plagioclase (Putirka 2008; Additional file 5: Fig. S4; Hartley and Thordarson 2013). The Mg# for olivine equilibrium was calculated using (molar Mg/[Mg + Fe^2+^]) with an estimated Fe^2+^/Fe^Total^ of 0.8 (Hartley et al. 2017). The Mg# for pyroxene equilibrium was calculated using (molar Mg/[Mg + Fe^total^]). The Ca# for plagioclase equilibrium was calculated using (molar Ca/[Ca + Na]).

Equilibrium plots show that the olivine from aʻa flows are not in equilibrium with proximal glass compositions while olivine from pahoehoe are closer, yet still out of equilibrium in comparison to vent samples (Additional file 5: Fig. S4a). Pyroxenes are typically not in equilibrium with their respective glass compositions except for the occasional rim point analysis from the event 3 pahoehoe (Additional file 5: Fig. S4b). Plagioclase show similar patterns, cores from the vents and pahoehoe are in equilibrium with their respective glass compositions, while plagioclase from aʻa flows are not (Additional file 5: Fig. S4c). Plagioclase patterns are consistent with trends seen in the Neave and Namur (2022) model (Additional file 5: Fig. S4d). The disequilibrium of crystals in aʻa flows may be attributed to the type of glass analyzed. Glass from aʻa samples are the composition of trapped interstitial melt after cooling and crystallization within the lava. Glass from the vents and pahoehoe were likely cooled at a faster rate and may better represent the overall composition of the erupting melt. Olivine and plagioclase analyses are largely in equilibrium with vent glass compositions [Mg#(Fe^2+^) = 30 and Ca# = 65]; however, no pyroxene cores will lie within the equilibrium field or below it (Additional file 5: Fig. S4b). A filter for pahoehoe and vent glass compositions with > 4 wt.% MgO (Additional file 5: Fig. S4) was applied to better simulate a carrier melt composition (Mg#(Fe^Total^) = 32, Mg#(Fe^2+^) = 46, and Ca# = 67). However, this simulated carrier melt would have only pyroxene rim analyses from pahoehoe and aʻa flows to be in equilibrium. While olivines are in equilibrium with an inferred melt composition similar to vent glass, pyroxenes originated from a more evolved melt, with a composition of Mg#(Fe^Tot^) = 40.

### Thermometry

Using the Putirka (2005) plagioclase-liquid thermometer, vent plagioclase crystallization temperatures range from 1070 to 1090 °C. Event 1 aʻa lava temperatures were 1026–1050 °C, and subsequent aʻa from event 2 was 995–1037 °C. The final stage pahoehoe was 1056–1086 °C. Vent scoria had the highest temperatures of 1080 ± 9 °C. Temperatures calculated using the Putirka (2008) plagioclase-liquid thermometer minutely differs, with vent plagioclase crystallization ranges from 1107 to 1117 °C, event 1 aʻa lava at 1068–1072 °C, event 2 aʻa lava at 1025–1076 °C, and event 3 pahoehoe at 1092–1113 °C. The liquid-only thermometer (Putirka 2008) yields temperatures consistent with the prior thermometers, with vent temperatures ranging from 1092 to 1124 °C, event 1 aʻa lava at 976–1009 °C, event 2 aʻa lava 937–972 °C, and event 3 pahoehoe with a range of 1002–1172 °C. Finally, the clinopyroxene-liquid thermometer (Putirka 2008) resulted in one suitable crystal-liquid pair in equilibrium at the vents, with a calculated temperature of 1116 °C.

### Impact of resolution on textural identification

**Fig. 10:**
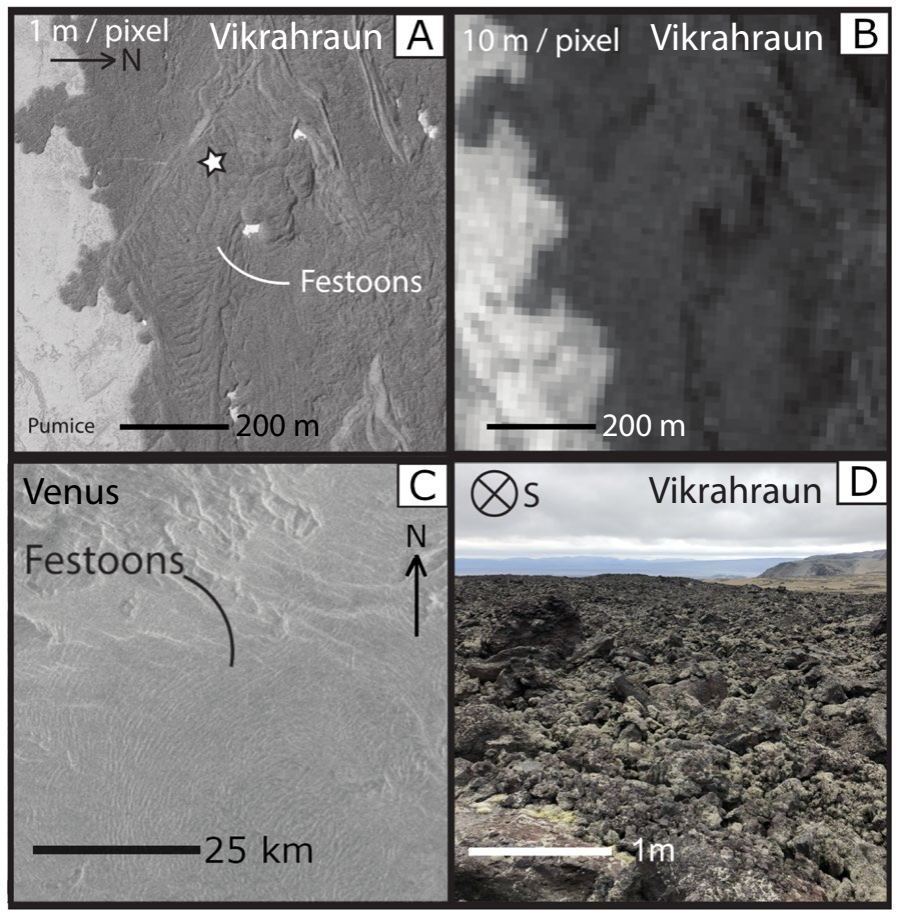
Lava surface features observed from a ground perspective and at varying overhead resolutions in comparison to the Ovda Fluctis Flow, Venus. **A** Greyscale aerial photo (taken by Loftmyndir ehf) at 1 m/pixel, with the white star showing the location of ground image D. **B** Optical image of the same area as A taken with the green band (band-3) from the Sentinel 2A satellite at 10 m/pixel resolution (Granule ID: L1C_T15TXG_A021473_20190802T170004). **C** Example of festooned flow on the northwest portion of Ovda Fluctis Flow, Venus, taken from Magellan SAR Left Look Mosaic (~ 75 m/pixel) and centered at -5.586°N, 95.242°E, and was retrieved from USGS Astropedia lunar and planetary cartographic catalog (https://astrogeology.usgs.gov). **D** Ground photo image from the event 2 aʻa flow on transect F. This location has festoons that are less than 1.5 m tall over a width of 10 m, but they are not evident from a ground perspective due to the uneven surface of aʻa blocks and clinker

Resolution plays a key factor in the interpretation of surface textures, especially when most data about planetary surfaces come from satellite imagery with typical resolutions of ~ 10–30 m/pixel. At these small-scale and coarse resolutions, identifying lava textures based on surface reflectivity would be possible, but only for an area equal to or larger than the pixel size (Fig. 10a and b). We recognize that delineation between pahoehoe and aʻa by surface reflectivity may also be affected by surface roughness, underlying slope, and weather conditions. However, the effects of these factors may be lessened by the selection of appropriate data and research location. Vikrahraun is an optimal location for satellite-based research as it has good contrast from the underlying topography, unchanging base slope in the direction of flow propagation, and minor vegetation. This leaves texture identification to be reliant on surface reflectivity and pixel resolution. Pahoehoe displays a higher reflectivity (pahoehoe ASTER Band-1 reflectivity: ~ 10.5%) at resolutions finer than 10 m/pixel, however at resolutions of 15 m/pixel, the average reflectivity decreases from more spectrally diffuse aʻa included in the pixel (aʻa ASTER Band-1 reflectivity: ~ 8%) (Additional file 5: Fig. S5) consistent with observations recorded by Brynes et al. (2004) at Kilauea Volcano, Hawai’i. The change in resolution causes pahoehoe to seemingly disappear and could cause the 1961 lava flow to be misinterpreted as having a higher overall viscosity.

Finer resolutions more common in terrestrial aerial photos and satellite images may highlight features that are not easily observable at the ground surface. For example, certain features such as lava flow levees or textural transitions from pahoehoe to aʻa on the scale of 2–5 m (Fig. 9e) are more obvious, while features such as aʻa festoons, which are on the scale of ~ 10 m in horizontal length, were not apparent on topographic profile traverse C (Fig. 9d). Festoons were not apparent here because the minimum resolution of height change on the Jacob’s staff was 1.5 m, so the 1 m tall crest height of the festoons in the event 2 aʻa middle lobe were not recorded (Fig. 9c; Fig. 10d). However, transects C and F (Fig. 9c and e) did confirm that the festooned area was composed of aʻa clasts (Fig. 10d) and would have been otherwise easily misinterpreted as large-scale pahoehoe ropes based solely on satellite and air photo imagery, even with a ‘high’ resolution of 1 m/pixel. These ground-truth observations are critical to properly identify such features and create a powerful analytical tool when used with satellite images (Fig. 10a).

### The 1961 Askja lava as a planetary analog

One of the significant findings of this study is the recognition of festoon ridges, which are downslope pointing convex ridges composed of surface lava clasts. Festoons also have been referred to as pressure ridges, ogives, and surface folds (Andrews et al. 2021). Documentation of terrestrial festoons are also unstandardized, describing a variety volcanic textures such as: small-scale pahoehoe ropes (Wentworth and MacDonald 1953; Larson 1991; Hon et al. 1994); large-scale flood basalt ribs, ridges, or folds (Fink 1980); large-scale convex ridges of aʻa clasts on Rangitoto Island, Auckland (Lowe et al. 2017) and in the 2018 Sierra Negra Eruption, Galápagos, Ecuador (Soule et al. 2019); large-scale convex andesitic and dacitic ridges called flow “wrinkles” in the young Gassan edifice, Japan (Oizumi et al. 2018); and large-scale convex rhyolitic flow ridges on the Obsidian Flow at Glass Mountain, California and on the Rocche Rosse Flow of Lipari, Italy (Fink 1983; Bullock et al. 2018; Andrews et al. 2021). Interestingly, rhyolitic festoons are similar in size and distribution to the festoons observed in the 1961 Askja flow, despite the difference in composition (Table 3).

**Table 3:** A summary of average festoon dimensions found on Venus, Earth, and Mars from cited literature

Location	Crest height	Wavelength	Arc length	References
Vikrahraun, Askja, Iceland	~ 5 m	~ 10 m	~ 150 m	This study
Obsidian flow, Glass mountain, CA, USA		~ 39 m	~ 250 m	Fink, 1980
Roches Rossa flow, Lipari, Italy	~ 15 m	~ 15 m	~ 150 m	Bullock et al. 2018
Odvo Fluctis, Venus		~ 1000 m	~ 50 km	Wroblewski et al. 2019
NW of Olympus Mons, Mars	~ 100 m	~ 180 m		Fink, 1980
North of Ascraeus Mons, Mars	~ 30	~ 50 m	~ 1 km	Zimbelman, 1985
SW of Arsia Mons, Mars	~ 27 m	~ 100 m		Warner and Gregg, 2003

Crest height refers to the festoon’s amplitude, wavelength refers to distance from one crest to the next, and arc length refers to the total width of the festoon

Terrestrial festoons have been used as analogs in interpreting lava flows found on Venus and Mars. Ovda Fluctis, on Venus, is a flow field that contains convex ridges with spacing of about 0.5–1.5 km between ridges (Fig. 10c) (Table 3) (Wroblewski et al. 2019). Studies suggested that the flow dimensions are reminiscent of rhyolitic lava flows (Schenk and Moore 1992; Permenter and Nusbaum 1994; McColley and Head 2004) and conclude that any similarity to pahoehoe is due to attributes of surface roughness and reflectivity. However, the overall morphology of the lobes and ridges and the dimensional fractal analysis indicate that the Ovda Fluctis flows are more likely basaltic (Wroblewski et al. 2019).

On Mars, festoons are observed in lava flows adjacent to major volcanic provinces and are also on a large scale (Table 3) (Fink 1980; Theilig and Greeley 1986, 1987; Crown and Ramsey 2017). The festoons are of two varieties: one occurring at distal dispersed flood basalt lobes (Theilig and Greeley 1986) and the other within channelized lava flows (Crown and Ramsey 2017). The rheologic constraints of the channelized festoons have been studied using ridge dimension and distribution (Fink 1980; Zimbelman 1985; Warner and Gregg 2003). Festoon flows north-west of Olympus Mons (Fink 1980), north of Ascraeus Mons (Zimbelman 1985), and southwest of Arsia Mons, Mars (Warner and Gregg 2003) (Table 3) each yield calculated estimated viscosities and display surface textures indicative of evolved flows. However, as demonstrated in this study of a mafic lava flow in Askja, the presence of festoons alone is not enough to imply a silicic composition. Therefore, previous assessments of silicic lava composition based on the presence of festoons should be re-evaluated.

## Conclusions

The repeated cycling from aʻa to pahoehoe as seen in the 1961 eruption at Askja, Iceland, has often been regarded as unconventional for a basaltic lava flow (Pinkerton and Sparks 1978). However, small-scale analyses of crystallinity, vesicularity, density, whole-rock chemistry, and mineral chemistry all indicate that the textural evolution is a reflection of changing eruption and emplacement conditions and is a predictable result of the eruption progression. Here, total crystallinity shows distinct trends between lava textures with increasing distance from the vents: aʻa flow crystallinity increases at 1% per km while pahoehoe flows increase in crystallinity with distance at higher rate of 6% per km. Vesicularity of all lava samples are systematically decreasing with increasing distance from the vents at a rate of ~ 1.4% per km. These trends indicate that pahoehoe or aʻa emplacement is largely controlled by temperature, which in the 1961 eruption is affected by differences in insulation between lava tubes and open channels. This insulation lowered the overall viscosity by about 10^2^ Pa s and caused the eruption to transition from emplacing aʻa to pahoehoe on Nov. 5, 1961.

The topographic profiles taken perpendicular to flow direction revealed temporal and overlaying relationships between various flow textures and their associated eruptive events. Among the features recorded most prominently in these profiles are aʻa levees which constrain unique flow features such as the festoons and show the evolution of the flow architecture, allowing for flow volume estimation and rheologic calculations (Fig. 9).

This study has demonstrated the value of the Vikrahraun lava as a planetary analog through the evaluation of the architectural components at various spatial scales in remote sensing images, compositional homogeneity, and the presence of mafic pressure ridges. Studies that use the presence of festooned lava flow to conclude that lava is of rhyolitic composition is an example of over-reliance on satellite data, which is one of the largest and easily accessible sources of extra-terrestrial information. The preference towards the analysis of large-scale surface features causes a neglect of more in situ analog studies that furthers the correlation of large-scale to small-scale textures that impact the rheologic controls of a lava flow. This study advocates for more test-bed studies to be conducted—among them the 1961 lava flow of Askja, Iceland.

## Supplementary Information

**Additional file 1**: Contains sample locations, an analytical summary containing a table of contents for Additional files 1-3, topographic profile data, and flow volume calculations, crystallinity, vesicularity, density, whole-rock chemistry, mineral chemistry, and additional calculations

**Additional file 2**: Textural data presented in this paper, including crystallinity, vesicularity, density, and viscosity calculations

**Additional file 3**: Cumulative chemistry data presented in this paper, including whole-rock chemistry, mineral chemistry, thermometry, and compiled geochemistry of Vikrahraun from the literature

**Additional file 4**: A detailed methodology section outlining each analytical dataset individually.

**Additional file 5**: Contains supplementary Fig. S1–S5 and associated captions

## Data Availability

All data generated or analyzed during this study are included in this published article and its supplementary information files.

## Abbreviations

‘phh’: Pahoehoe
‘PB’: Pahoehoe breakout
‘S. Thor’: Sigurđur Thorarinsson
‘pl’: Plagioclase
‘cpx’: Clinopyroxene
