# Supplementary material for: Vikrahraun—the 1961 basaltic lava flow eruption at Askja, Iceland: morphology, geochemistry, and planetary analogs
Source: Earth Planets Space. 2022 Nov 12;74(1):168. doi: 10.1186/s40623-022-01711-5 (PMC9653356; doi:10.1186/s40623-022-01711-5)
Supplement: Supplementary file 4 — Additional file 4: A detailed methodology section outlining each analytical dataset individually. [file 40623_2022_1711_MOESM4_ESM.docx]

*Field methods-sample collection*

Three field seasons were conducted in 2015, 2018, and 2019 in which a total of 39 rock samples and 6 topographic profiles were collected. A summary of sample locations and texture may be found in figure 1 and Additional file 1.1. We also provide another table that identifies which analyses were performed on each sample (major and trace element ICP-MS and XRF analysis, mineral and glass EMPA analysis, thin section, crystallinity by ImageJ, porosity, permeability, density by pycnometer, and calculations of viscosity and thermometry) in Additional file 1.2. Most samples were collected from the top surface of the lava flow, except for samples IIAU19-005 and IIAU15-016 which were collected from an internal part of the event 3 pahoehoe flow. A total of 6 topographic profiles trending N-S range in length from 0.2 km – 1.1 km, and totaling ~4.5 km, span the flow from near-vent (4 km) to the central section (5 km) to distal (8 km) (Figure 1c). Topographic profiles were completed in teams, with measure tape and laser range finder and Jacob’s Staff. The Jacob Staff’s level was set to 1.5 m height and each 1.5 m increment elevation increase or decrease was spatially located with GPS and measured in distance from the prior position by tape measure and laser range finder**.** Topographic profiles consist of 16 to 134 measured points, with a maximum change in elevation across the profile of ± 5.5 m. Profiles were imported into ArcMap © and overlain on SPOT satellite imagery (2.5 m/pixel). All lava textural features such as rubbly a’a or shelly pahoehoe were noted and marked on the profiles as well as more localized features such as armored lava balls, levee walls, kipukas, lava tubes, and ponding structures.

*Methods for Flow Volume Estimate*

GPS coordinates of sample and topographic profile locations were plotted in ArcMap© using the SPOT satellite imagery (2.5 m/pixel) (Figure 1b) found in the ESRI World Basemap layer (ESRI, 2019). Greyscale aerial photo taken by LoftMyndir ehf (1.0 m/pixel) (Figure 1c) and WorldView-2 satellite (1.8 m/pixel) imagery were also used to create a visual classification map delineating a’a and pahoehoe surfaces into polygons based on the characteristics of pahoehoe having a smooth highly reflective surface and a’a having a rough and weakly reflective surface (Byrnes et al., 2003; Crown and Ramsey, 2016) (Figure 2). This general surface textural map was then divided into flows representing emplacement in each of the three eruptive events based on 1961 eruption observations by Thorarinsson and Sigvaldason (1962) (Figure 1c). The second eruptive event is also further divided into event 2 a’a and event 2 pahoehoe. The eruption map was then sub-classified into 5 flow lobes that are defined as the north lobe (N Lobe), middle north lobe (Mid N Lobe), middle south lobe (Mid S Lobe), the south lobe (S Lobe), and the western halo (W Halo) (Figure 1c)**.**

Topographic profiles were corrected for basement elevation change by calculating an estimated line slope of the 1875 Holocene pumice surface for each transect, which may be viewed in Additional files 1.3- 1.8. Elevations were measured in the field with GPS, taking data from off-lava surface location endpoints of transects and, when present, from kipukas. The interpolated surfaces were subtracted from the topographic profile elevations to get an estimate of thickness for each eruptive event at each transect. Individual event volumes were then calculated by multiplying flow area by the average flow thickness (Additional file 1.9).

*Petrography and Crystallinity*

Mineral textural characterization and crystallinity estimates were conducted on each thin section. Minerals smaller than 0.1 mm were excluded from the phenocryst abundance estimations and were considered to be part of groundmass crystallinity. A total of 24 backscatter images were acquired from the Hitachi S-3400 Scanning Electron Microscope at the University of Iowa on the same 8 thin section samples that were analyzed by EPMA for mineral and glass compositions. For each sampled thin section, three separate areas of groundmass were targeted to reduce the influence of the variability found in the groundmass fabric and imaged with magnification ranging from 75 to 130 with a 15 kV acceleration voltage. Backscatter images were processed on ImageJ to estimate modal abundances of microlite minerals in the groundmass using the threshold selection tool to analyze the areal percent of a specific mineral phase in thin section. Reproducibility was assessed by conducting 3 separate iterations per image, yielding an average standard deviation (1σ) of 3.5%. Data is present in Additional file 2.1 and 2.2.

*Vesicularity*

Thin section scans were collected on 33 of the samples and measured with the Modeling Object Structure & Analysis Information Calculator (MOSAIC) (Moritz in prog). This is a software application designed to produce quantitative morphometrics and model structures for geological digital images. It focuses on vesicle objects using the open source computer vision image processing library OpenCV. MOSAIC applies blob detection algorithms using spatial proximity and pixel threshold calculations to determine “in” vs “out” vesicle designations based on probability distributions consistent with a Laplacian of gaussian context filter. MOSAIC reports metrics of circularity, convexity, compactness, roughness, roundness and sphericity as well as area, volume, symmetry, and vesicularity quantities. The reproducibility of vesicularity and average area was assessed by measuring the sample 3 times and adjusting the “erosion factor” or the crispness of the vesicle edge with each iteration. The average standard deviation (1σ) is 1.5% for vesicularity and 0.18 mm for vesicle sizes. Data is presented in Additional file 2.3.

*Density and Connected Porosity*

Bulk and matrix densities and connected porosity was measured for all 38 samples at the University of Auckland, New Zealand. using a micrometrics Geopyc 1360 and Accupyc 1340. Bulk density was determined with the Geopyc by measuring the displacement of DryFlo beads to calculate sample volume that includes interstitial space of a sample. Matrix density was measured with the Accupyc, measuring the volume of displacement of He gas. Connected porosity was then calculated by taking the difference between the bulk volume and the matrix volume divided by bulk volume and multiplied by 100. Reproducibility was assessed by taking 3 measurements with each instrument per sample and taking the average volume measurements for density calculations and were typically on the order of 0.3 g/cm^3^ for bulk density and 0.1 g/cm^3^ for matrix density. Data is present in Additional file 2.4.

*Whole-rock major and trace elements*

Whole-rock major and trace element data was measured with the Thermo X-series Inductively Coupled Plasma Mass Spectrometer (ICP-MS) at the University of Iowa following the procedure outlined by Peate et. al (2010) and Reagan et. al (2013). All 39 samples from 2015 and 2018 were crushed, powdered with porcelain ball mills into ~100 mg portions per sample, and digested in a mixture of HNO_3_ and HF. The solutions were diluted and evaporated twice with HNO_3_ and a third time with HCl. The final evaporates were dissolved in an HF-HCl solution and finally diluted with HNO_3_ for analysis. Data was calibrated with standards W-2, BIR-1, BHVO-2, BCR-2, AGV-2, JA-1, BRP-1. Reproducibility and data quality are assessed with the basalt standard W-2. Which may be viewed in Additional file 3.1. Accepted values for the elemental abundances in the SRMs can be found at: <http://georem.mpch-mainz.gwdg.de/>. The relative standard deviation for major and trace element composition are < ± 2 %, with the exception of P_2_O_5_, Ni, Zn, Nb, Mo, Cd, Sn, Ta and W based on replicate analyses of the W-2 standard. (see Additional file 3.1).

Samples collected in 2019 were analyzed using an 193 nm Excimer Laser Ablation ICP-MS for major element and a Thermo Scientific iCAPQ ICP-MS for trace element concentrations at Michigan State University. Sample preparation and XRF analyses follow procedures outlined by Rooney et al. (2012). Reproducibility was assessed with standards BHVO-1, JB1A, and RGM-2 analyzed as unknowns, and have a relative standard deviation < ± 0.13 % with the exception of Na_2_O with a r.s.d. of 0.6% in the BHVO-1 (see Additional file 3.1). Trace element reproducibility was assessed with standards BHVO-1 and JB1A and have a r.s.d. < ±0.05 % except for Ni with a r.s.d. of 0.1% in BHVO-1 and 0.08% in JB-2.

*Mineral Chemistry*

A total of 8 samples were analyzed with the JEOL JXA-8230 Electron Microprobe Analyzer (EMPA) at the University of Iowa for mineral phase compositions of olivine, plagioclase, pyroxene, and glass. The samples are representative of the entire lava flow, with one from the vent area (IIAU18-006), one from the event 1 a’a (IIAU18-001), three from the event 2 a’a (IIAU14-013, IIAU15-022, IIAU15-023), one from the event 2 pahoehoe (IIAU15-012), and 2 from the event 3 pahoehoe (IIAU19-004 and IIAU19-008) (Figure 1B and C). Standard operating conditions of 15 kV, and a 10 nA beam current of 3 μm diameter were used. Minerals standards used were Astimex olivine, Astimex chrome pyrope, Astimex apatite, Smithsonian augite, and Smithsonian plagioclase, BCR-2G, ATHO-G1, and VG-568. Standard reproducibility was assessed with these mineral and glass standards and is <0.5 wt.% (2σ) for all elements except for SiO_2_ and Na_2_O in glass analyses which have 2σ of 0.6 and 1.6 wt.%, respectively (see Additional files 1.15 – 1.18 for details). Data quality was assessed using the stoichiometric totals of minerals. Values calculated to be 100 ± 2% and cation sums ± 0.1 of the accepted value were considered appropriate for use. Olivine results were based on 10 analyses of 6 crystals from the vents, 26 analyses of 20 crystals from event 1 a’a, 21 analyses of 16 crystals from event 2 a’a, 10 analyses of 8 crystals from event 2 pahoehoe, and 24 analyses of 19 crystals from event 2 pahoehoe. Plagioclase results were based on 28 analyses of 24 crystals from the vent, 20 analyses of 20 crystals from event 1 a’a, 67 analyses of 63 crystals from event 2 a’a, 33 analyses of 28 crystals from event 2 pahoehoe, and 37 analyses of 38 crystals from event 3 pahoehoe. Pyroxene results were based on 19 analyses of 15 crystals from the vents, 42 analyses of 21 crystals from event 1 a’a, 92 analyses of 62 crystals from event 2 a’a, 28 analyses of 23 crystals from event 2 pahoehoe, and 41 analyses of 48 crystals from event 3 pahoehoe. Glass results were based on 10 analyses from the vents, 11 analyses from event 1 a’a, 4 analyses from event 2 a’a, and 15 analyses from event 3 pahoehoe. Data is presented in Additional files 3.2 – 3.6.

*Satellite and Drone Data*

Sentinel-2A L1C data was acquired by the sensor on August 2, 2019. ASTER L1B data was acquired by the sensor on August 7, 2012), both were retrieved from USGS Glovis (<https://glovis.usgs.gov/>) with cloud cover under 10%. All datasets were projected in Transverse Mercator in UTM zone 27 on the WSG datum.

Drone imagery was collected during the summer of 2019 with a Mavic 2 Pro Camera equipped with a 1' CMOS passive sensor. Images were taken at nadir and oblique perspectives capturing a 400 m wide 1400 m long footprint of the lava at a resolution of 0.08 m/pixel. The data was calibrated with three 1 x 1.6 m flags as ground controls points and was used to produce a DEM with the stereophotogrammetry software Agisoft Metashape Professional.

***References***

Moritz, AJ (2022 in prog) Quantitative Morphometrics of Vesicular Samples using Open Source Software Methods: Implications for 2 and 3-D image analysis of Geologic Materials. ProQuest Dissertations and Theses database.
