# Supplementary material for: Vikrahraun—the 1961 basaltic lava flow eruption at Askja, Iceland: morphology, geochemistry, and planetary analogs
Source: Earth Planets Space. 2022 Nov 12;74(1):168. doi: 10.1186/s40623-022-01711-5 (PMC9653356; doi:10.1186/s40623-022-01711-5)
Supplement: Supplementary file 5 — Additional file 5: Contains supplementary Fig. S1–S5 and associated captions [file 40623_2022_1711_MOESM5_ESM.docx]

**Additional File 5**


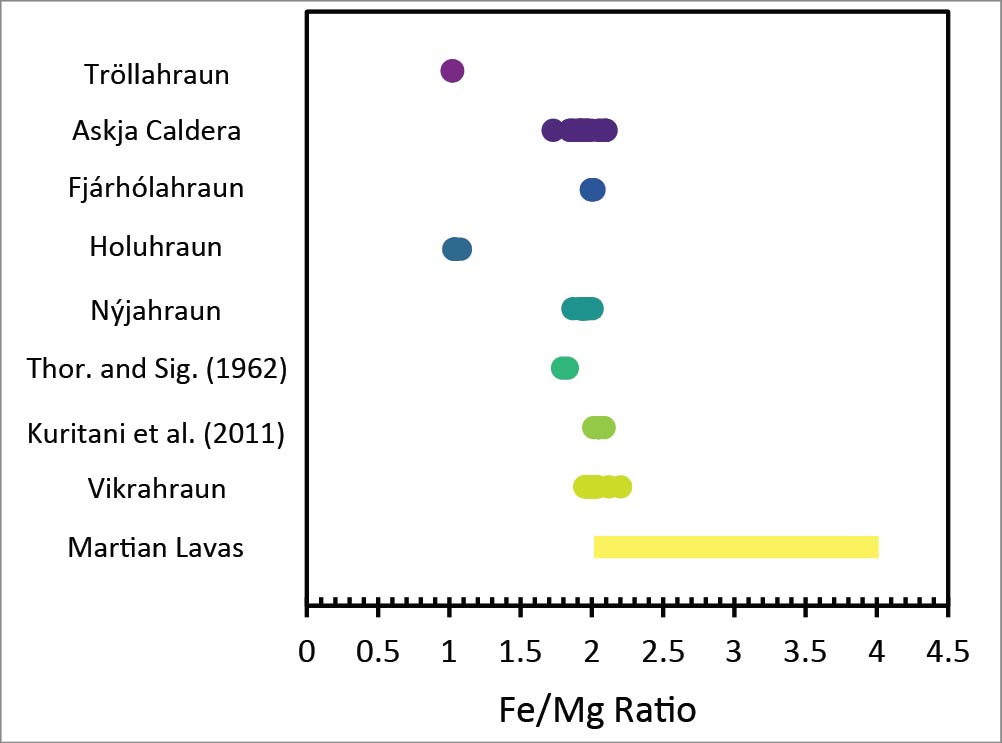


**Fig. S1**

A comparative geochemical plot between the Fe/Mg ratios of martian lavas (McSween 2009) and various Icelandic lavas from the Askja volcanic system, Tröllahraun of the Veiðivötn volcanic system, and the 2015 Holuhraun eruption (from Hartley and Thordarson 2013 and Geiger et al. 2016). Included are previously published geochemical data from the 1961 eruption (Thorarinsson and Sigvaldason 1962 and Kuritani et al. 2011).


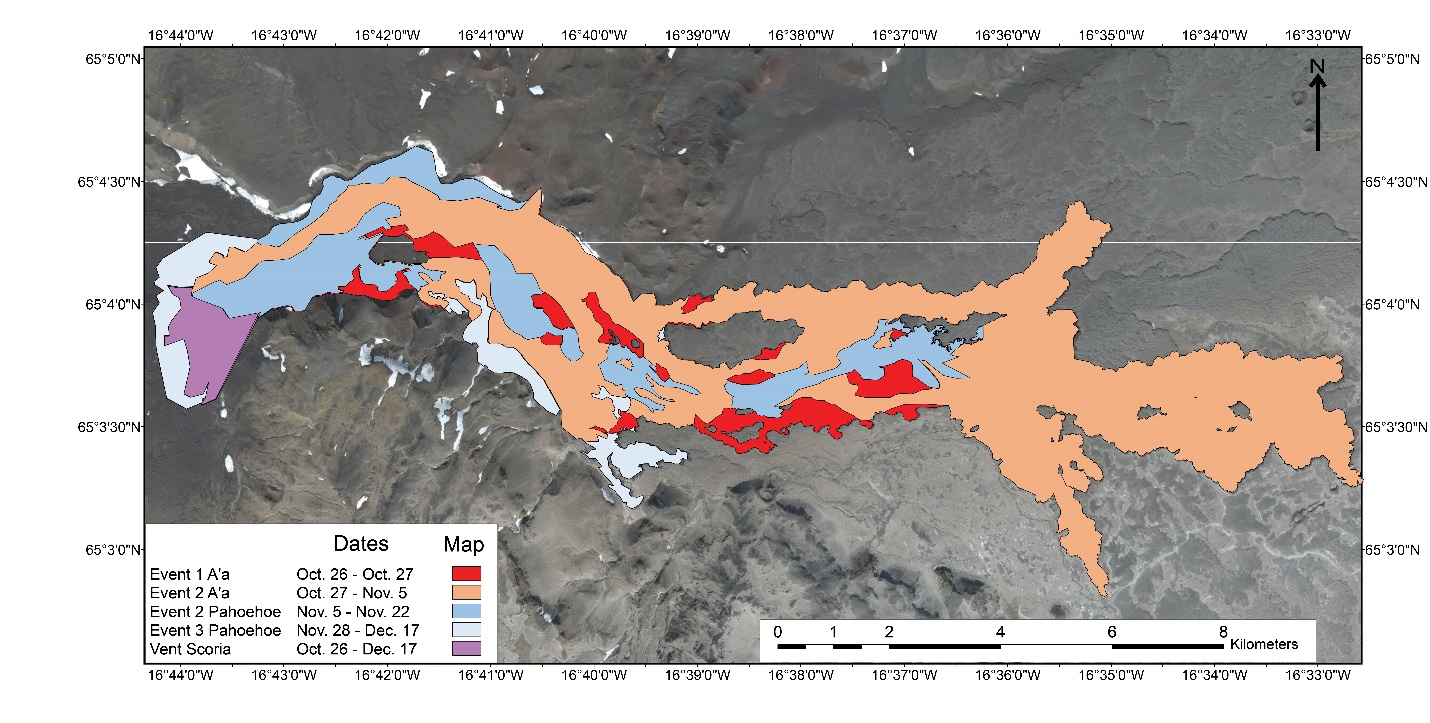


**Fig. S2**

A simplified, colored map of the 1961 Vikrahraun eruption.


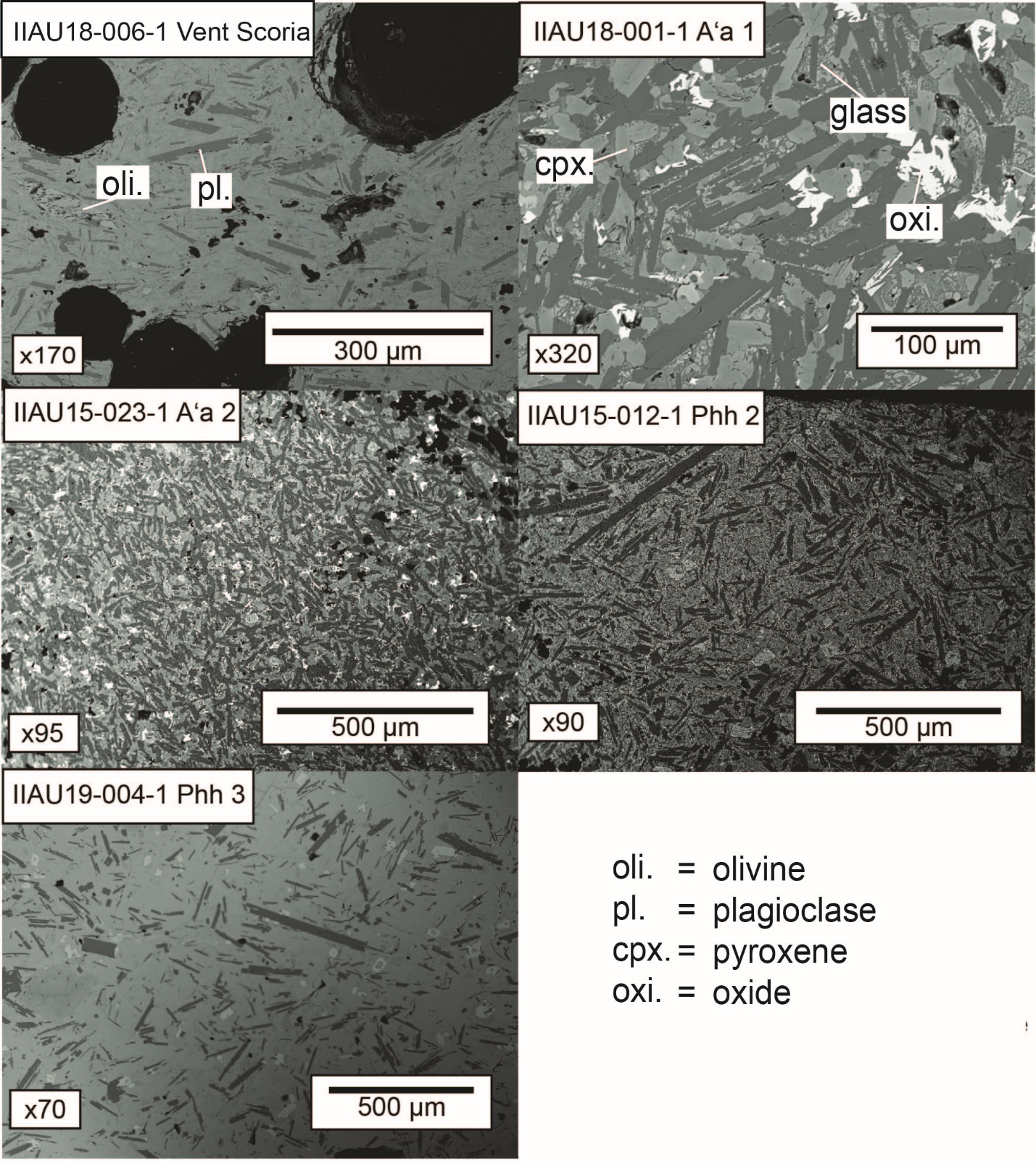


**Fig. S3**

Representative backscatter images of each eruptive event used to calculate total sample crystallinity. Vent scoria and event 1 aʻa images have labelled crystals to exhibit the brightness difference between minerals. This allows for a selection by brightness threshold on imageJ to calculate the relative abundance of minerals. Olivine is abbreviated to ‘oli.’, plagioclase to ‘pl.’, clinopyroxene to ‘cpx.’, and oxide to ‘oxi’.


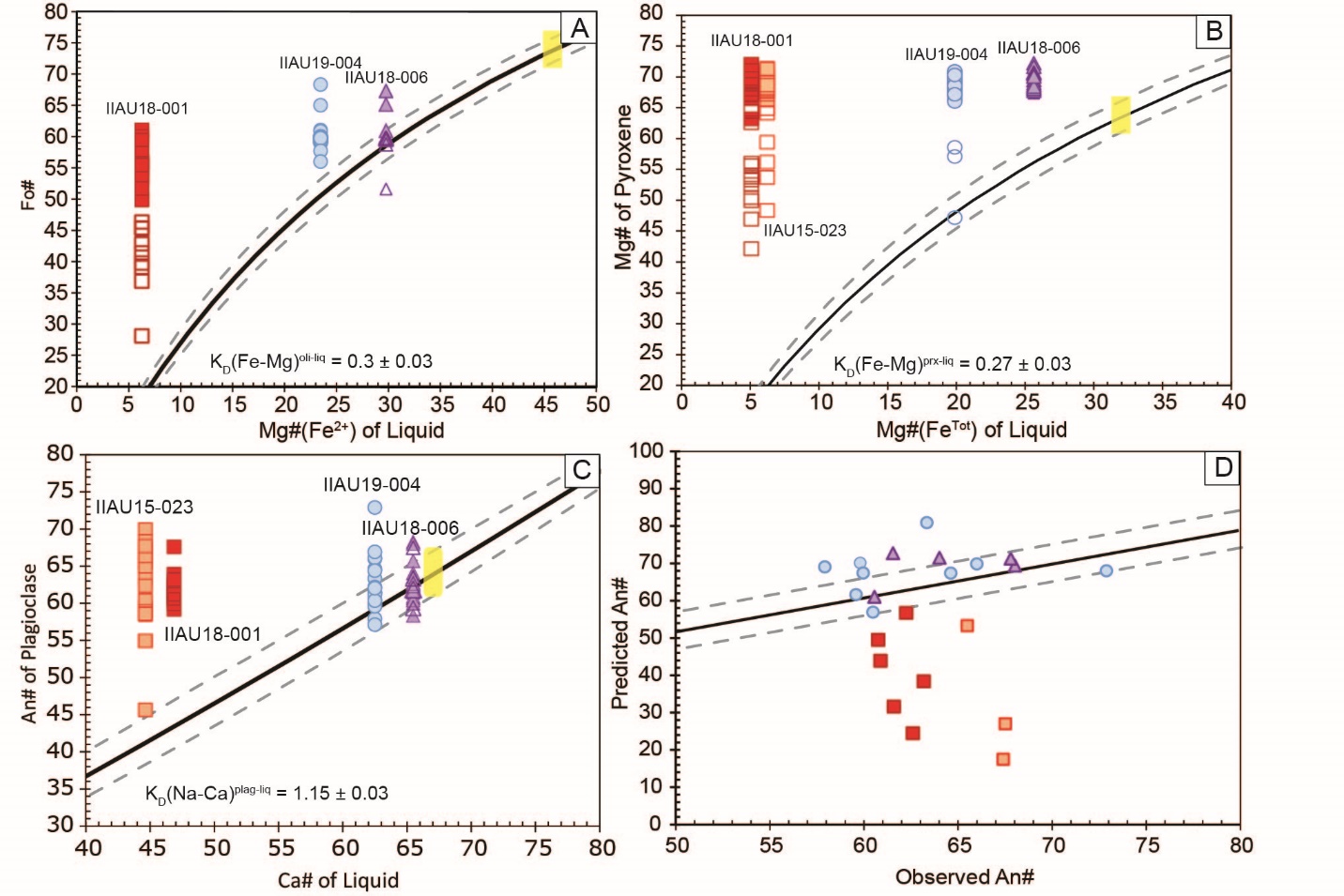


**Fig. S4**

The Rhodes diagram, following Roeder and Emslie (1970), Putirka (2008), and Neaves and Putirka (2017) is used to determine equilibrium between mineral-melt pairs. Open symbols correspond to crystal rim analyses and closed symbols correspond to crystal core analyses. The lines represent the Kd field and is equivalent to 0.3 ± 0.03 for olivine (Kd Fe-Mg (oli-liq)), 0.27 ± 0.03 for pyroxene (Kd Fe-Mg (oli-liq)) and 1.15 ± 0.03 for plagioclase (Kd Na-Ca (plag-liq)). The yellow field corresponds to the composition of crystals in equilibrium with a hypothesized carrier melt (see text for details). A) Mg# of the liquid (molar Mg / [Mg + Fe^2+^]) vs the Fo# (molar Mg / [Mg + Fe^2+^]) show that olivine crystals from aʻa and pahoehoe flows are in disequilibrium with their associated glass compositions. However, if we assume that olivine crystals from the lava flow crystalized from the likely erupting lava composition at vents Mg#(Fe2+) = 30, then their ranges would be overlapping with the equilibrium field. Olivine would be in disequilibrium with a hypothesized carrier melt of Mg#(Fe^2+^) = 46 B) Mg# of the liquid (molar Mg / [Mg + Fe^Total^]) vs the Mg# show that pyroxenes are not in equilibrium with their associated glass composition and were likely accumulated. Even if Mg# of the liquid for all samples had glass compositions similar to the vents (Mg#(Fe^Tot^) = 25) or a carrier melt of Mg#(Fe^Tot^) = 32, disequilibrium in most pyroxene crystal cores would still be present. C) Ca# of the liquid (molar Ca / [Ca + Na]) vs the An# (molar Ca / [Ca + Na +]) show aʻa plagioclase crystals to be out of equilibrium with their associated glass compositions. If we assume plagioclase crystallized from glass compositions from the vents (Ca# = 65) or a carrier melt (Ca# = 67), aʻa plagioclase would plot largely within the equilibrium field. E) A plagioclase equilibrium model after Neave and Namur (2022) plots predicted An# of plagioclase from glass compositions compared to observed An#. Samples that lay within the 95% prediction interval are in equilibrium.

**
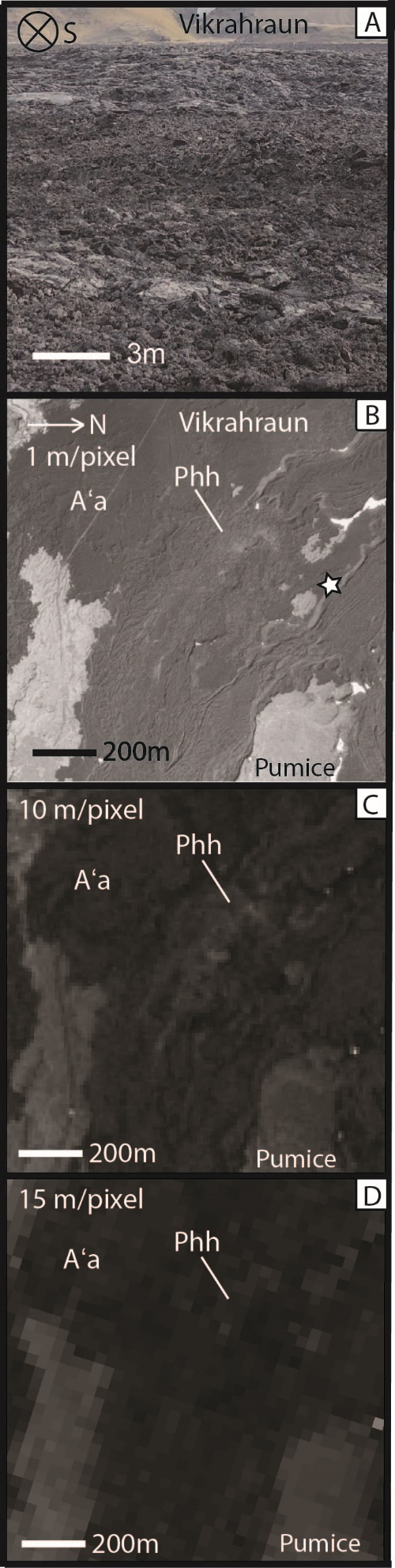
**

**Fig. S5**

Pahoehoe and aʻa surface textures observed from a ground perspective and at varying overhead resolutions from Sentinel 2-A (Granule ID: L1C_T15TXG_A021473_20190802T170004) and ASTER L1B (Granule ID: AST_L1B_00308112010123826_20101015073517_23000) satellites. A) A ground photo image taken from the end of transect A showing the juxtaposition of event 2 blocky aʻa and shelly pahoehoe textures. B) Greyscale aerial photo (taken by Loftmyndir ehf) at 1 m/pixel resolution with the white star showing the location of ground image A. C) Optical image of the same area taken with the green band (band-3) from the Sentinel 2A satellite at 10m/pixel resolution. D) Optical image of the same area taken with the green band (band-1) from the ASTER satellite at 15m/ pixel resolution.
